# cGAS–STING axis: A central regulator of neural homeostasis and neuroinflammatory pathogenesis

**DOI:** 10.4103/NRR.NRR-D-25-00367

**Published:** 2025-08-13

**Authors:** Jiajie Zhang, Jiarui Li, Yanan Li, Chunxiao Liu, Lei Shi, Yuxuan Qian, Qian Chen, Qi Zhang

**Affiliations:** 1 Graduate School of Hebei Medical University, Shijiazhuang, Hebei Province, China; 2 Department of Anesthesiology, Hebei Children’s Hospital, Shijiazhuang, Hebei Province, China; 3 Department of Anesthesiology, The Third Hospital of Hebei Medical University, Shijiazhuang, Hebei Province, China; 4 Department of Orthopedic Surgery, Sixth People’s Hospital Affiliated to Shanghai Jiao Tong University School of Medicine, Shanghai, China

**Keywords:** astrocytes, cGAS–STING axis, microglia, mitochondrial DNA, neurodegeneration, neuroimmunology, neuroinflammation, neurological disorders, NLRP3, small molecule inhibitors, type I interferon

## Abstract

An increasing amount of evidence shows that type I interferon response, which is induced by cyclic guanosine monophosphate-adenosine monophosphate synthase (cGAS) and stimulator of interferon genes (STING) is closely associated with health and neuroinflammatory diseases. Abnormal activation or loss of control of the cGAS–STING axis affects the development of neuroinflammation. Thus, we examined its role in major neurological diseases, including traumatic brain injury, Alzheimer’s disease, Parkinson’s disease, Huntington’s disease, multiple sclerosis, herpes simplex encephalitis, and ataxia-telangiectasia. Additionally, targeted intervention of the cGAS–STING axis to control neuroinflammation and treat related diseases has become the focus of current clinical research. This article describes the development of cGAS inhibitors and small molecules that target the cGAS–STING axis and explores the potential applications of STING inhibitors and agonists in clinical research. In summary, the cGAS–STING axis may impact neurological diseases more than a single protein or gene. Future studies should focus on elucidating the functional dynamics and regulatory networks of this axis and delineating its crosstalk with other signaling cascades. These investigations will provide mechanistic insights for developing targeted therapeutic strategies for associated disorders and potentially facilitate drug repurposing across diverse disease contexts.

## Introduction

Cyclic guanosine monophosphate-adenosine monophosphate (cGAMP) synthase (cGAS) and stimulator of interferon genes (STING) are being intensively researched as important neuroinflammatory mediators (Liu et al., 2024b). The cellular cGAS–STING axis plays an essential role in the immune system. This axis recognizes the existence of cytoplasmic DNA and triggers the expression of inflammatory genes (Maekawa et al., 2019; Ning et al., 2020). As the core components of this axis, cGAS and STING are pivotal in recognizing DNA abnormalities within the cell and participating in the immune response against pathogens, thus regulating and maintaining normal immune system function (Zhang et al., 2020). As a recently discovered immunomodulatory mechanism, cGAS–STING axis has involved considerable insight from researchers working in neuroinflammatory regulation (Chen et al., 2022b). cGAS–STING signaling orchestrates mitochondrial DNA (mtDNA)-driven interferon (IFN)-β production through TANK-binding kinase 1 (TBK1)-IFN regulatory factor 3 (IRF3) phosphorylation (Hu et al., 2021b; Ouyang et al., 2023), which induces the production of cytokines such as CXC chemokine ligand 10, interleukin (IL)-6, and tumor necrosis factor-α (TNF-α). Emerging evidence demonstrates that pharmacological modulation of cGAS–STING axis suppresses microglial NOD-, LRR- and PYD-containing protein 3 (NLRP3) inflammasome activation in lipopolysaccharide-induced neuroinflammatory models (Decout et al., 2021). STING promotes microglial polarization towards the pro-inflammatory phenotype while suppressing immunosuppressive microglial polarization through the downstream axis, including IRF3 and nuclear factor-κB (NF-κB) (Kong et al., 2022). Collectively, these measures reduce overexuberant neuroinflammation and enhance the prognosis of individuals with cerebral vascular diseases (Zhao et al., 2021). However, a previous study has also demonstrated the potential of cGAS–STING axis to exert neuroprotective effects by enhancing synaptic plasticity (Huang et al., 2025). This article will review how progress in cGAS–STING axis research has contributed to the understanding of neuroinflammatory diseases. In addition, we discuss how the axis could be modulated for the therapies of neuroinflammatory conditions.

## Search Strategy

To comprehensively evaluate the research progress on cGAS–STING axis in neuroinflammatory diseases, this study adopts a systematic literature search strategy to ensure coverage of significant achievements and the latest developments in the field.

### Database selection

The search scope includes the following core biomedical databases: PubMed (biomedical literature database), Web of Science (multidisciplinary high-impact journal coverage), Scopus (comprehensive abstract and citation database), and Embase (biomedical and pharmacological literature database).

### Keyword and search query construction

Combining MEdical Subject Headings (MeSH) terms and free-text terms, the following keyword combinations were designed:

Core Concept 1: cGAS–STING Signaling axis

Related terms: “cGAS–STING axis” OR “cyclic GMP-AMP synthase” OR “stimulator of interferon genes” OR “cGAS/STING axis.”

Core Concept 2: Neuroinflammatory Diseases

Related terms: “neuroinflammatory diseases” OR “neuroinflammation” OR “Alzheimer’s disease” OR “Parkinson’s disease” OR “multiple sclerosis” OR “traumatic brain injury” OR “viral encephalitis.”

Boolean Operator Combination: (Core Concept 1) AND (Core Concept 2]).

### Timeframe and screening criteria

Timeframe: January 2020 to January 2025 (focusing on the past 5 years, with an emphasis on the most recent 3 years to meet timeliness requirements).

Language restriction: Only English-language literature was included.

Literature types: Priority was given to original research, reviews, and meta-analyses; conference abstracts, commentaries, and irrelevant studies were excluded.

### Search and screening process

Initial screening: A total of 2350 articles were retrieved from the databases, and 1820 articles remained after removing duplicates.

Title/abstract screening: Based on relevance to the research topic, 420 potentially relevant articles were identified.

Full-text assessment: After downloading and reviewing the full texts, 187 articles were ultimately included, with 54% (101 articles) published in the most recent 3 years (2022–2025).

### Data extraction and analysis

The following information was extracted from the included literature: (1) Activation mechanisms and functional roles of cGAS–STING axis in different neuroinflammatory diseases; (2) Interactions between the axis and other inflammatory signaling axes (e.g., NLRP3 inflammasome, Toll-like receptor (TLR) axis); (3) Advances in preclinical and clinical studies on targeted intervention strategies; and (4) Content analysis was used to integrate the data, with a focus on mechanistic studies, contradictory findings, and consensus within the field.

### Screening and analysis of literature

A comprehensive literature search was conducted using PubMed, Web of Science, Embase, and the Cochrane Library, covering the period from inception to May 2025. This search employed keyword combinations such as “cGAS–STING signaling axis,” “cGAS–STING axis,” “cyclic GMP-AMP synthase,” “stimulator of interferon genes,” “cGAS/STING axis,” in conjunction with terms such as “neuroinflammatory diseases,” “neuroinflammation,” “Alzheimer’s disease,” “Parkinson’s disease,” “multiple sclerosis,” “traumatic brain injury,” and “viral encephalitis.” The retrieved literature was further supplemented by manual reference checks. The inclusion criteria focused on original research (cellular, animal, or clinical) investigating the mechanisms or interventions of the cGAS–STING axis in the context of neuroinflammation, while excluding irrelevant topics and reviews. Following a thorough screening of titles, abstracts, and full-text evaluations, all pertinent references were included. Data extraction encompassed models, activating factors, pathways, and intervention effects, which were categorized by disease type. Quality assessment was performed using the SYRCLE scale for animal studies and the NOS scale for clinical studies, with the findings synthesized through thematic analysis.

### Quality control

Cross-validation (two authors (JZ and JL) screened the literature) was used to reduce bias, with disputes resolved by a third researcher (QZ). Manual searches of references from highly cited reviews were conducted to ensure coverage of key historical studies.

## cGAS–STING Signal Transduction Axis

### Basic structures of the cGAS and STING proteins

Being a cytoplasmic DNA sensor, cGAS can trigger STING protein which further induces immune response by generating cGAMP (Sun et al., 2013; Wu et al., 2013). STING is also believed to control various disease states involved in the inflammatory response and oxidative stress (Ablasser et al., 2013). The unique structure of cGAS allows it to recognize mislocated DNA in cells, and then provoke a relevant immune response. cGAS is a member of the nucleotidyltransferase protein family (nucleotidyltransferase domain), two fundamental domains of cGAS are able to bind DNA and perform a transfer of nucleotidyl. The N-terminus of cGAS consists of a functional region harboring a highly conserved sequence of 130–150 amino acid residues which functions in sensing double-stranded DNA (dsDNA) in cells, and is central to the proteins to detect aberrant DNA. The central, nucleotidyl transferase motif forming the catalytic domain is the major site of function for cGAS, which is where cGAS produces the DNA agonist cGAMP. cGAMP production triggers other types of immune responses, such as activating the STING protein, which subsequently produces an immune response. The C-terminal Mab21 domain in cGAS is important not only for its catalytic activity and binding of the dsDNA, but also for the structural study that showed this domain can undergo conformational changes after binding to dsDNA and is critical for cGAMP synthesis (Civril et al., 2013; Sun et al., 2013). Significantly, a putative highly conserved zinc finger is at the C-terminus of cGAS, and zinc ions for protein maintenance of structural stability. The zinc finger domain serves as an important recognition site for cGAS to identify dsDNA. Its inactivation affects the subsequent correct binding and recognition of dsDNA by cGAS, which will deteriorate its essential biological roles.

STING is an endoplasmic reticulum or Golgi apparatus-localized adaptor protein, responsible for mediating the innate response to bacterial products. It is expressed by epithelial and endothelial cells, macrophages and dendritic cells (Ishikawa and Barber, 2008). STING is composed of a short N-terminal cytoplasmatic segment, four helices, a cytoplasmatic ligand-binding domain, and a C-terminal tail (Ouyang et al., 2023). Its transmembrane and ligand-binding domains engage in a specific dimerization termed the “domain-swapper.” The cGAMP engages the central opening of the dimerized ligB domains that leads to a set of concerted changes in the downstream conformation of the dimer termed the rotation of the “wings” of the domain-dimer inward toward the N-terminal domain, and closure of the protein binding site. The minimal structure of STING could be described into the following: (1) a transmembrane region formed by four transmembrane helices (TM1–TM4) that link STING to the cell membrane; (2) a cytoplasmic ligand binding domain associated with cGAMP and other ligands, which triggers conformational modifications and homodimerization of STING that leads to the activation of downstream pathways; (3) a C-terminal tail where we note that this region is also implicated in signaling interactions and generally harbors a PXPLRXD motif, where X may represent any amino acid residue. The motif is essential for the recruitment of TBK1. Once TBK1 is bound to the motif, it phosphorylates targets, particularly S366 in the cytoplasmatic tail of STING, which is the essential trigger of a cascade of molecular actions that includes the recruitment and later phosphorylation of the transcription factor IRF3. Phosphorylation of IRF3 is essential for the induction of IFNI expression to trigger antiviral adaptive immunity in the host. Integrated functions of cGAS and STING are critical to the proper operation of the immune system (Ishikawa and Barber, 2008).

### Mechanism of cGAS–STING axis activation

**Figure 1:**
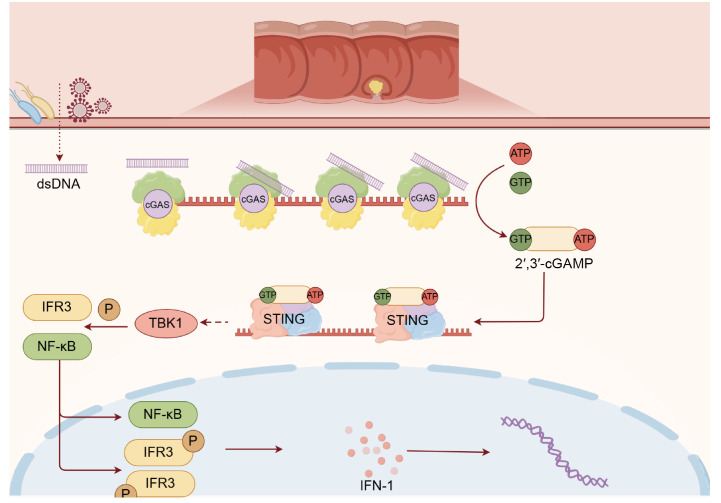
Mechanism of cGAS–STING axis activation. 2′,3′-cGAMP: 2′3′-Cyclic guanosine monophosphate-adenosine monophosphate; ATP: adenosine triphosphate; cGAS: cyclic guanosine monophosphate-adenosine monophosphate synthase; dsDNA: double-stranded DNA; GTP: guanosine triphosphate; IFN-1: type I interferon; IRF3: interferon regulatory factor 3; NF-κB: nuclear factor kappa-light-chain-enhancer of activated B cells; P: phosphorylation; STING: stimulator of interferon genes.

cGAS recognizes intracellular dsDNA, like atypical deposition of extraneous forms of DNA such as viral DNA, pathogenic bacterial DNA and instable genomic DNA. The genomic DNA can be accumulated in cytoplasm, in the absence of multiple nuclease enzymes (Gao et al., 2015). DNA can also enter cytoplasm in different ways (Chamilos et al., 2012; Klarquist et al., 2014; Schoggins et al., 2014), which is then sensed by cGAS to trigger cGAS–STING signaling cascade. Upon recognition of DNA, the enzyme cGAS stimulates the formation of cGAMP from adenosine triphosphate and guanosine triphosphate and thereby up-regulates the expression of the STING gene (Ge and Ding, 2022). Further, cGAMP binds and activates STING in the cell within which it is formed or in a neighboring cell through binding to volume-controlled anion channels or gap junctions (Domizio et al., 2022). As an adaptor protein, STING bridges the interaction between upstream cGAS and downstream TBK1 to activate the IRF and NF-κB axis, inducing the upregulation of type I interferons (IFN-I) and some inflammatory cytokines (CXC chemokine ligand 10, TNF-α, and IL-6). The activation of the STING axis enhances the synthesis of IFN-I, particularly IFN-α and interferon-beta (IFN-β), through the transcription factors IRF3 and NF-κB. Interferons (IFNs) are pivotal cytokines in the immune system’s response, mediating a range of processes from antiviral defense to the induction of autoimmunity (Chin, 2019). STING signaling axis activation can additionally enhance the activation state of natural killer and T cells (Nicolai et al., 2020; Lu et al., 2023), which contributes to heightening the amplitude of the immune response (**Figure 1**).

## Cell-Specific Regulation of cGAS–STING Axis

### Role of cGAS–STING axis in microglia

#### Activation and function of microglia

Microglia serve as the key immune cells in the central nervous system (CNS) and monitor the environment of the CNS by responding to injury, pathogen, or infection (Yang et al., 2025). The activity of these immune cells can be triggered by different axes such as infection, injury, or neurodegenerative diseases. Microglia are at rest in the normal physiological condition where they help with neuronal survival, neuronal health, and aiding neural network formation. Nonetheless, with the pathological conditions, microglia are morphologically and functionally activated, releasing inflammatory factors, such as TNF-α and IL-1β, and inducing a neuro-inflammation (Li et al., 2024a).

The activation of microglia is not just reflected by morphological transition, but with many prominent changes in gene expression. Activated microglia can strengthen phagocytosis, eliminate debris and pathogen, as well as recruit other immune cells by releasing pro-inflammatory factors to form local inflammatory responses (Zhu et al., 2021a). However, over-activation or its continuous activation may become chronic neuroinflammation and further induce neuronal death and neurological dysfunction will become important pathological mechanism of various neurodegenerative disorders, including Alzheimer’s disease (AD) and Parkinson’s disease (PD) (Liu et al., 2021).

Microglial functions are not immune-related only: they are involved in neurodevelopment, synaptic pruning and neuroprotection (Bohlson and Tenner, 2023). It is known that microglia contribute to the robustness of neural networks during neurodevelopment by pruning redundant synapses and ensuring neuron survival (Bai and Liu, 2021). Hence, mechanisms of microglia activation and their participation in the neuroinflammation process are of urgent importance for the development of therapy targeting neurological diseases.

#### Specific role of cGAS–STING axis

cGAS–STING axis is an important mechanism for microglia in responding to intracellular DNA, including pathogen DNA and self-DNA. cGAS acts as a DNA sensor that can recognize dsDNA in the cytoplasm and synthesize cGAMP, which subsequently activates STING, leading to a robust immune response, including the production of IFN-I (Ke et al., 2022).

In microglia, the activation of cGAS–STING axis not only promotes the release of inflammatory factors but also regulates the functional state of microglia. Research has found that the excessive activation of cGAS–STING axis is closely related to the progression of various neuroinflammatory diseases, such as in models of AD (Chung et al., 2024; Zhang et al., 2024a). Abnormal activation of cGAS–STING axis is considered an important factor leading to persistent inflammatory responses in microglia and neuronal damage (Chen et al., 2021). In addition, cGAS–STING axis is also involved in the autophagy process of microglia, regulating the intracellular inflammatory response, which further affects the survival and function of microglia (Schmid et al., 2024).

Thus, we note that the regulation of the cGAS–STING axis has cell-specific properties: the cGAS activation in microglia represents novel functional properties specific to that of other cell lineages, e.g., macrophages. For example, nerve injury can activate the cGAS–STING axis in the microglia leading to conversion to pro-inflammatory state in microglia but other lines of cells may provoke distinct immune response in other cells (Shi et al., 2024b). Cell-specificity of this also underlines the importance of cGAS–STING axis as a drug target of choice for understanding the mechanism of neuroinflammation and linked disorders.

In summary, the specific role of cGAS–STING axis in microglia includes both the promotion of inflammatory responses and the regulation of cellular physiological functions. The research on this axis provides an important theoretical foundation for understanding the mechanisms of neuroinflammation and developing new therapeutic strategies.

### Regulation of cGAS–STING axis in neurons

#### Specific response of neurons to inflammatory reactions

Neurons play a unique role in inflammatory responses, particularly in neuroinflammatory diseases, where the activation of cGAS–STING axis is closely related to neuronal responses. cGAS acts as an intracellular DNA sensor that can recognize dsDNA in the cytoplasm and activates the STING signaling axis by synthesizing cGAMP. Thereby inducing the production of IFNs and other inflammatory factors. This process has been confirmed to play an important pathological role in various neurodegenerative diseases, such as AD and PD (Kumari et al., 2024; Liu et al., 2024a). The neuronal activation of cGAS–STING axis has a close relationship with intracellular DNA released as well as cellular stress response. It was shown that pathological environmental conditions including hypoxia and oxidative stress can destabilize the mitochondria membrane and lead to the abnormal release of mtDNA into the cytoplasm and activation of the cGAS–STING axis. This can lead to continuous inflammation in the microglia and astrocytes of the CNS. Abnormal neuroinflammatory microenvironments not only cause apoptosis, such as neuronal apoptosis and pyroptosis, but also are highly related to the pathological process of some neurodegenerative diseases, such as AD and PD (Gąssowska-Dobrowolska et al., 2024; Hu et al., 2024). Activation of the cGAS–STING axis is identified as a key trigger of neuronal injury and dysfunction in the ischemic brain injury model (Chauhan and Kaundal, 2023a, b). In addition, neuronal induction of inflammation is closely related to interactions with the major CNS immune cells, microglia. Generally, activation of microglia is correlated with induction of the cGAS–STING axis. In the AD model, the cGAS–STING axis in microglia is suggested to be important in amyloid-beta (Aβ) plaque removal whereas its activation in neurons aggravates the inflammation and causes neuronal cell death (Kumari et al., 2024; Liu et al., 2024a). All in all, we suggest that neuronal proinflammatory response is regulated through activation of the cGAS–STING axis affecting neuronal survival and activity. This mechanism, not only gives insights into the pathogenesis of neuroinflammation but also gives target candidates for the therapy of neuroinflammatory disease.

#### Regulatory mechanism of cGAS–STING axis

The regulation of the cGAS–STING axis is complicated, which is subject to multiple-level regulations, such as transcriptional regulation, protein interaction, and intracellular signal transduction. As a DNA sensor, cGAS will be affected by the intracellular situation, especially cytoplasmic DNA amount and nature (Sun and Hornung, 2022; Zhang et al., 2024a; Chen et al., 2025). Studies have demonstrated cGAS can detect exogenous pathogen DNA and endogenous mtDNA and activate the STING signaling axis and produce IFNs and pro-inflammatory factors (Chauhan and Kaundal, 2023a, b; Kumari et al., 2024).

In neurons, cGAS is also regulated by various factors different from its catalytic interaction with DNA. For example, different proteins including poly(rC)-binding protein 2 (PCBP2) were revealed to be negative regulators for cGAS activity, which were achieved by restraining the proper cGAS compatible condensation, as well as ensuring the appropriate production of cGAMP and balanced status of the cGAS–STING axis (Gu et al., 2022). In addition, both cGAS compatible condensation and phase separation are important in modulating its activity and both processes are prone to be modulated by intracellular metal ions (e.g., Mn^2+^) and other biomolecules (Hu et al., 2023; Shinde and Li, 2024).

STING activation is also negatively or positively regulated by multiple mechanisms. Intracellular trafficking and oligomerization of STING are critical for efficient signal propagation. It has been shown that the STING trafficking from endoplasmic reticulum to Golgi apparatus is essential for its activation and aggregation depending on the binding of cGAMP and mediated by other endogenous signaling molecules (Hu et al., 2023; Shinde and Li, 2024). The induction of STING is closely connected with the intracellular autophagy mechanism, which has two opposing functions: controlling the cGAS–STING axis and maybe reducing pathological inflammatory reaction by phagocytosing cell damaged DNA (Schmid et al., 2024; Shinde and Li, 2024).

In summary, the cGAS–STING axis in neurons, in short, is a circuit consisting of multi-directional interaction among many intracellular signals, external environment signals, or communication between cells and outside. Understanding the circuit helps both to reveal the neuroinflammation pathogenesis and to pinpoint possible targets for neuroinflammation therapy. In the future, studies on specific roles and regulation mechanisms of the signaling axis of cGAS–STING in different neurologic diseases should continue for a new concept and strategies for clinical treatment.

### Role of astrocytes in neuroinflammation

#### Functions of astrocytes and inflammatory responses

Astrocytes, which make up about 90% of the glial population in the CNS, have numerous crucial roles among others sustaining the neuronal metabolic homeostasis, mediating synapse development, controlling the blood–brain barrier (BBB) permeability and taking part in neuroinflammation. They promote the survival and differentiation of neurons by secreting neurotrophic factors and cytokines and participate in the regulation of neural injuries and diseases (Won et al., 2025).

In the period of neuroinflammation, astrocytes would participate in a set of functional remodeling: morphological and gene expression, which is typically termed as the reactive astrocytes that are capable of secreting different pro-inflammatory as well anti-inflammatory factors (e.g., TNF-α, IL-1β, and IL-6). These released cytokines in addition to mediating local inflammation process, also facilitate neuronal damage and death (Chauhan and Kaundal, 2023b; Kumari et al., 2024).

Astrocytes also exert two-edged functions on neuroinflammation: they have an ability to inhibit inflammation and serve as neuroprotection by producing anti-inflammatory factors, whereas the overexcited astrocytes could amplify the inflammatory response and aggravate the destruction of neurons (Paul et al., 2021; Liu et al., 2024a). This functional conversion has a close relationship with the polarization of the astrocytes: generally, astrocytes polarize into A1 type with pro-inflammatory properties, and A2 type with a protective function (Guo et al., 2024).

Both are capable of enhancing NF-κB signaling and prolonged signaling in inflammation can inhibit IRF3 to control excessive immune-stimulation. Finally, feedback mechanisms such as A20 and suppressor of cytokine signaling are capable of shutting down signals through repression of STING or inhibition of TBK1 to allow for homeostasis. Such balance is of special interest as excessive activation may promote tumor growth via reactive oxygen species or inflammation pathway (Li et al., 2021).

#### Duality of pathology and therapy

cGAS–STING axis mediates immune defense and pathological damage through IRF3 and NF-κB, respectively. For instance, diABZI-induced PANoptosis (a hybrid of apoptosis, pyroptosis, and necroptosis) can eliminate infected cells (Li et al., 2021; Wei et al., 2024), but the released mtDNA may secondarily activate cGAS–STING, forming an inflammatory amplification loop that leads to ARDS. In cancer treatment, it is essential to balance STING activation to enhance immune responses while avoiding excessive inflammation; whereas in autoimmune diseases, inhibiting STING can mitigate tissue damage (Zhou et al., 2023; Guo et al., 2025).

In summary, cGAS–STING axis orchestrates immune and inflammatory responses through IRF3 and NF-κB, and its precise regulation is pivotal for future antiviral, antitumor, and inflammatory disease therapies.

### Interaction between cGAS–STING and the NLRP3 inflammasome

#### Synergistic mechanism

cGAS–STING axis, upon recognizing cytoplasmic DNA within cells, can initiate a series of immune responses, particularly by inducing the production of IFNs and proinflammatory cytokines to enhance the body’s defense against pathogens (Zheng et al., 2020; Liu et al., 2022). Recent studies have demonstrated a significant synergistic interaction between cGAS–STING axis and the NLRP3 inflammasome (Shi et al., 2024a; Yang et al., 2024; Bai et al., 2025). The NLRP3 inflammasome is a crucial intracellular sensor capable of recognizing various pathogens and endogenous danger signals, such as DNA release caused by cellular damage. By activating caspase-1, it promotes the maturation and secretion of IL-1β and IL-18 (Zhu et al., 2021a).

When the cGAS–STING axis is activated, intracellular cGAMP serves as a second messenger to promote the assembly and activation of NLRP3. It is indicated that the interplay between cGAS and NLRP3 not only enhances NLRP3 activity but also aids in the formation of inflammasomes by mediating NLRP3 positioning of NLRP3 to the endoplasmic reticulum (Zhang et al., 2023b). Moreover, activation of the cGAS–STING axis leads to the activation of transcription factors including NF-κB and IRF3, which in turn boost the expression level of NLRP3-related pro-inflammatory factors. This generates a positive feedback loop and magnifies the inflammatory response (Bai and Liu, 2021).

#### Antagonistic mechanism

Despite the synergistic effects between the cGAS–STING axis and the NLRP3 inflammasome, it is shown that antagonistic connections may take place on certain occasions. There is evidence showing that the excessive activation of the cGAS–STING axis will regulate the dysregulation of intracellular inflammation which would trigger autoimmune disorders or chronic inflammation. For instance, uncontrolled activation of the cGAS–STING axis has been strongly associated with the pathogenesis of autoimmune diseases such as systemic lupus erythematosus and rheumatoid arthritis (Li et al., 2024a).

Similarly, activation of the NLRP3 inflammasome could be attenuated by the inhibitory pathway of the cGAS–STING axis. Indeed, a previous report has revealed that an antagonist of NLRP3 (such as IL-10), which suppresses NLRP3, and reduces inflammation via downregulation of the cGAS–STING axis (Chen et al., 2021). In addition, the NLRP3 inflammasome induction can also decrease the cGAS–STING axis in a negative feedback manner in order to regulate excessive inflammation response to a certain extent (Gaidt et al., 2017).

#### Impact on inflammation and autoimmune diseases

The interplay between the cGAS–STING axis and the NLRP3 inflammasome is pivotal in the pathogenesis of various inflammatory and autoimmune disorders. Evidence suggests that the concurrent activation of the cGAS–STING axis and the NLRP3 inflammasome synergistically facilitates the initiation and progression of these diseases (Litwiniuk et al., 2021; Han et al., 2023; Carnazzo et al., 2025). Indeed, there is functionally interplay between cGAS–STING axis and NLRP3 inflammasome in response to cell stress/damage, the two work in positive feedback via mtDNA release and oxidative stress, and further enhance inflammatory signal (Li et al., 2023; Shi et al., 2024a). For instance, in patients suffering from systemic lupus erythematosus, the cGAS–STING axis exhibits dysregulated activation in association with the activation of the NLRP3 inflammasome that leads to high levels of proinflammatory cytokine secretion (including IL-1β and IL-18), contributing to the amplification of the inflammatory response of the disease (Liu et al., 2021).

Furthermore, the cGAS–STING axis and NLRP3 inflammasome interplay and are closely associated with tumor-associated inflammation. In tumor microenvironment, cGAS–STING axis activation could induce NLRP3 inflammasome activation and enhance tumor-associated inflammation and influence tumoral initiation/progression (Zhu et al., 2021a). Thus, manipulating the connection between the cGAS–STING signaling circuit and the NLRP3 inflammasome might provide new implications and approaches for the treatment of inflammatory/autoimmune disorders.

### Interrelationship between cGAS–STING and TLR axis

#### Downstream effects of common signal transduction

The signaling axis of cGAS–STING and that of TLR has complex interaction, and each has a fundamental impact on immune response. As an intracellular DNA sensor, cGAS can detect dsDNA in the cytoplasm and stimulate STING to make cGAMP, which then mediates downstream signaling axes such as expression of IFN-I and other proinflammatory cytokines (Ke et al., 2022). The TLR axis regulates mainly immunological responses through the identification of pathogen-associated molecular patterns and damage-associated molecular patterns. Recently it has been found that the cGAS–STING and TLR signaling pathways show cross-regulatory roles in the transduction of intracellular signaling, especially during infection and inflammation (Lio et al., 2016; Danielson et al., 2024). For instance, TLR7 and TLR9 can sense viral RNA and DNA, thereby triggering downstream NF-κB and IRF signals that stimulate IFN-I production (Chen et al., 2021). At the same time, cGAS–STING axis activation can enhance the TLR signaling pathway activation leading to positive feedback to amplify the immune response. Apart from improving host antimicrobial defenses, this interaction could also lead to excessive inflammation in some cases that could lead to autoimmune diseases (Danielson et al., 2024).

#### Cell type–specific responses

The cGAS–STING and TLR pathway crosstalk displays the cell type–dependent specificity. In plasmacytoid dendritic cells, the cGAS–STING axis activation leads to inhibition of TLR9-induced interferon production. cGAS–STING axis activation has been demonstrated to feedback inhibit TLR9 activity through the suppressor of cytokine signaling, which partially restricts excessive stimulation of the TLR pathway signaling (Zhu et al., 2021a). This process plays a vital role in viral infection and autoimmune disease since it participates in the regulation of the immunity homeostasis, and inhibition of the excessive immune response.

Additionally, the role of the cGAS–STING axis in hepatocytes, macrophages and dendritic cells is not uniform. The cGAS–STING axis is likely associated with the clearance of hepatitis viruses in hepatocytes. However, in macrophages, the cGAS–STING axis may regulate the tumor microenvironment by influencing proinflammatory activities (Li et al., 2024b). The different cell-type specific response exemplifies the critical importance of accounting for cell type–specific activities and responses of cGAS–STING and TLRs when developing therapeutic options.

#### Bidirectional regulation of anti-infective and tumor immunity

Bi-directional feedback regulation between the cGAS–STING axis and TLR pathways plays an essential role in the modulation of the immune response against infections and tumorigenesis. In infection, cGAS–STING axis is triggered by the detection of pathogen-associated DNA, which induces production of IFN-I and other pro-inflammatory cytokines, resulting in an antiviral immune response (Li et al., 2024a). Parallel to the TLR pathway, the activation of immune cells and inflammation through the recognition of pathogen-associated molecular patterns constitutes an important dimension of immune mechanisms which simultaneously adds strength to the fight against diseases (Wicherska-Pawłowska et al., 2021; Danielson et al., 2024).

The cGAS–STING axis in the tumorigenesis microenvironment has complex effects. On one hand, cGAS–STING signals activate to help the tumor immune response and stimulate T cell activity to suppress tumors (Wang et al., 2024). On the other hand, tumor cells can avoid immune surveillance to avoid tumors by downregulating the cGAS–STING axis process and promote tumor (Bai and Liu, 2021). Such a bidirectional regulatory relationship suggests that investigating the crosstalk of cGAS–STING axis and TLR pathways should be an important research direction in tumor immunotherapy studies.

To sum up, the cGAS–STING and TLR pathways may participate as regulators in the infection and tumor immunity. Further exploration of the interaction between the two axes the cGAS–STING and TLR pathways will offer a fundamental theoretical basis and experimental reference for the potential use of new immunotherapeutic regimens.

### Collaboration and antagonism between cGAS–STING and AIM2 axis

#### Role of AIM2 and its interaction with cGAS–STING axis

IM2 (absent in melanoma 2) serves as an important cytoplasmic, intracellular DNA sensing molecule, which senses dsDNA in the cytoplasm, and induces inflammation via the assembly of AIM2 inflammasome complex. AIM2 activation triggers the release of cytokines and pyroptosis, which eliminates virus-infected or damaged cells (Zhu et al., 2021b; Yu et al., 2024). The cGAS–STING axis consists of cGAS as the main DNA sensor that is triggered upon detection in the cytoplasm of the dsDNA in the cytoplasm which results in the synthesis of the 2′,3′-cGAMP, thereby activating STING that initiates the downstream signaling cascades that lead to of IFNs and the secretion of inflammatory mediators. The coordination of cGAS–STING axis is crucial for immune responses, especially during viral infections and in the tumor microenvironment (Corrales et al., 2016; Chen et al., 2022a; Xu et al., 2024).

Research indicates that there exists a complex interplay between the AIM2 and cGAS–STING axis. In certain circumstances, the activation of AIM2 can enhance the signaling of cGAS–STING axis. For instance, when cells are infected by viruses or suffer DNA damage, the activation of AIM2 can promote the activation of cGAS, thereby enhancing IFN production and antiviral responses (Chen et al., 2021). However, excessive activation of AIM2 or cGAS–STING signaling may lead to chronic inflammation and the onset of autoimmune diseases (Xu et al., 2024; Yu et al., 2024; Yue et al., 2025), suggesting that we should consider the interaction between two axes in our research It is necessary to consider its dual role in different pathological states.

#### Role of cGAS–STING and AIM2 axis in cell death

cGAS–STING and AIM2 signaling pathways are important for cell death during cGAS–STING axis can enhance the cell immune responses to cause IFN and proinflammatory cytokine secretion, whereas AIM2 can induce pyroptosis or apoptosis via inflammasome assembly. It occurs as an immune defense mechanism against pathogenic infection and plays an important role in tumor microenvironment. It was found that AIM2 activation can induce pyroptosis through the assembly of the AIM2 inflammasome, which is a key cell death pathway responsible for tumor and infected cell death (Zhu et al., 2021a). Meanwhile, the cGAS–STING axis can induce cell death, especially during DNA damage and infection, it promotes cytokine release via the downstream activation of IRF3 and NF-κB transcription factors (Kuhl et al., 2023). Therefore, the interplay between the cGAS–STING and AIM2 pathways in regulating cell death evidently not only impacts cell viability but also significantly influences tumor immunosuppression and chronic inflammation.

#### Impact on T cell function

The activation of cGAS–STING and AIM2 signaling axes significantly impacts the functionality of T cells. Research has found that the activation of cGAS–STING axis can enhance the anti-tumor immune response of T cells, promoting the proliferation and cytotoxic response of CD8^+^ T cells (Ke et al., 2022). In the tumor microenvironment, the activation of cGAS–STING can enhance the activity and efficacy of T cells by promoting the production of IFNs, thereby increasing the killing effect on tumor cells (Wang et al., 2023). The activation of AIM2 also impacts T cell function. AIM2 enhances T cell activation and proliferation by promoting the release of inflammatory factors, particularly in the contexts of infection and tumor environments The role of AIM2 may enhance the anti-tumor capacity of T cells by modulating the local microenvironment. However, excessive activation of AIM2 or cGAS–STING signaling may lead to T cell exhaustion and dysfunction, which is particularly evident in the tumor microenvironment, suggesting that we need to exercise caution when designing immunotherapeutic strategies based on these two axes. It is necessary to balance its activation level to avoid negative impacts on T cell function (Li et al., 2024a).

Overall, the cGAS–STING and AIM2 signaling axis plays intricate but powerful roles in regulating cell death, T cell function, and immune responses. This provides fundamental insights into their disease associations and reveals potential therapeutic opportunities.

## cGAS–STING Axis in Neuroinflammatory Diseases

Neuroinflammation is believed to cause the underlying neurodegeneration and neuronal loss in neurological disease (Zhang et al., 2025a). Neuroinflammation refers to the activation of the CNS induced by infection, toxic metabolites, trauma, and autoimmune factors. In neuroinflammation, microglia and astrocytes are activated and modulated by cGAS–STING axis producing nitric oxide, reactive oxygen species and proinflammatory cytokines, together to induce neuronal damage and dysfunction. Particularly, mtDNA released following neuronal injury activates cGAS, leading to the production of cGAMP and subsequent phosphorylation of TBK1 by STING. These subsequently induce the nuclear translocation of IRF3/NF-κB and the expression of inflammatory cytokines (Hu and Tian, 2024; Zhang et al., 2024b). A recent study has also shown that ARF1 mutations impair the recycling of STING by COPI vesicles, resulting in its pathological accumulation in the Golgi apparatus, and persistent activation IFN signaling axis, thus promoting brain inflammation (Hirschenberger et al., 2023). Conversely, pharmacological inhibition of cGAS (e.g., G140) or inhibition of ATP-binding cassette subfamily C member 1 (ABCC1) mediated cGAMP export significantly reduced neuroinflammation in ischemic stroke models (Hu and Tian, 2024; Shinde et al., 2025).

### Traumatic brain injury

Traumatic brain injury (TBI) is a traumatic effect or impact jolted to the head, or a penetrating wound to the skull with damage to the brain tissue (Wagner et al., 2021; Zhang et al., 2025b); is a dreaded condition of neurological disturbances that is followed by permanent or temporary loss of function (Freire et al., 2023). TBI is considered two phases, the primary (acute) mechanical injury, induced by any external forces and secondary injury, that is a result of second hit caused by possible complications such as hypoxia, ischemia and inflammation/cell death (Ogonah et al., 2025). Neuroinflammation is an important factor in the pathogenesis of neuronal death and neuronal dysfunction following TBI (Zheng et al., 2022). Fritsch et al. (2022) demonstrated that IFN-I signaling intensifies the early tissue injury caused by TBI. Moreover, IFN-I response is amplified through the NOD-like receptor X1 (NLRX1)-cGAS–STING signaling pathway. The cGAS/STING deficient mice show better tissue and functional outcomes at a short 14-day time post-TBI. Moreover, this study showed that NLRX1 functions as an inhibitor of STING activation in the brain, which means that NLRX1 would be an interesting target for therapeutic purposes. Meanwhile, this study examined mtDNA for the first time as an inducer of STING-IFN-I signaling following neuronal injuries.

Zhang et al. (2022) noted that upregulation of STING exacerbated neuroinflammation and neuronal injury after severe TBI. Conversely, STING antagonist C-176 appeared to be neuroprotective by blocking the NLRP3 pathway and accordingly attenuated neuroinflammation significantly attenuated.

**Figure 2:**
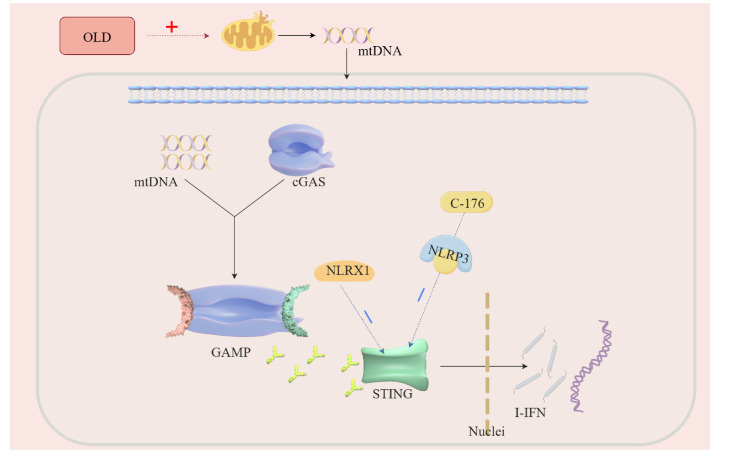
Role of cGAS–STING axis on TBI. TBI triggers mitochondrial damage, leading to the liberation of mtDNA, which subsequently activates cGAS–STING-IFN axis, thereby amplifying the neuroinflammatory reaction. In addition, NLRX1 and NLRP3 may negatively regulate STING activation in the brain and interfere with signal transduction. C-176: A STING antagonist; cGAS: cyclic guanosine monophosphate-adenosine monophosphate synthase; GAMP: guanosine monophosphate-adenosine monophosphate; IFN: interferon; I-IFN: type I IFN; mtDNA: mitochondrial DNA; NLRP3: NOD-, LRR- and PYD-containing protein 3; NLRX1: NOD-like receptor X1; OLD: oxidized mitochondrial DNA; STING: stimulator of interferon genes; TBI: traumatic brain injury.

In a preclinical trial from Barrett et al. (2021), the authors noted that after age-associated TBI, elderly animals also exhibit higher expression of interferon-associated genes and enhanced cGAS activation contributing to the stronger neuroinflammatory response. Such results are consistent with clinical trial findings that reported that neuroinflammation manipulating interventions (e.g., stimulation of the right median nerve) have improved long term outcome, and thus hold some therapeutic targets for TBI (Sharp and Graham, 2023; **Figure 2**).

Lastly, we can conclude that neuroinflammation is an essential step in neuronal death and dysfunction in TBI pathogenesis and evidence in this field has been comprehensively revealed molecular mechanism inducing neuroinflammation onset and progression in TBI and offered definite guidelines for therapeutic approaches. Targeting STING activation, for example, NLRX1 or employing STING inhibitors to dampen inflammatory signaling axis provides new treatment options for TBI that has high clinical relevance in terms of helping to optimize long-term patient health and well-being, and also sets a good basis for further theory-guided research into treatment options for TBI.

### Alzheimer’s disease

AD is a common form of neurodegenerative disease with degeneration of the brain and consequent cognitive impairments and memory loss (Xie et al., 2023). Neuroinflammation plays an important and central role in the pathogenesis of AD (Kinney et al., 2018). Among AD patients, ongoing immune responses occur in the brain tissues with long-lasting activation of microglia and other immune cells. As the key intrinsic immune cells in the CNS, the microglia release a number of pro-inflammatory cytokines such as IL-1β, IL-6 and TNF-α, when activated in AD. These cytokines can aggravate Aβ and tau pathologies, which leads to a vicious cycle that increases neuroinflammation and neuronal damage (Webers et al., 2020). Secondly, the aggregation of Aβ and hyperphosphorylation of tau proteins can act as DAMPs that activate PRRs on microglia to trigger an inflammatory response (Cai et al., 2022; Chen and Yu, 2023; Sun et al., 2024b). Furthermore, neuroinflammation is closely associated with the cognition deficits observed in AD patients, with studies demonstrating a direct association between the presence of inflammatory markers and the degree of cognitive dysfunction (Contreras et al., 2022).

The accumulated evidence has established a strong link between cGAS–STING axis to the pathogenesis and progression of AD. cGAS–STING axis is abnormally activated in AD brains and may mediate AD via multiple pathways. On the one hand, the activated cGAS–STING axis may induce neuroinflammatory responses to cause pathological changes of Aβ and tau proteins. For example, IFN-I, which is produced via the cGAS–STING axis, can amplify inflammatory responses of microglia to release more proinflammatory cytokines, which aggravate the neuroinflammation and neuronal injury (Govindarajulu et al., 2023). On the contrary, this signaling pathway can also lead to neuronal injury and death, studies show that inhibiting of cGAS–STING axis can reduce neuronal damage and improve cognitive performance (Yan et al., 2025). However, the pathway’s effects are complex, depending on cell types and disease stages, requiring further mechanistic investigation.

Neuroinflammation plays a key role in the epidemiology of AD. Studies show increased concentrations of diverse inflammatory markers like TNF-α, IL-9, IL-12p40 and so on in the cerebrospinal fluid of AD patients and an increased concentration of such markers to be correlated with an enhanced probability of getting AD (Contreras et al., 2022). As example, elevated baseline levels of TNF-α, IL-9, and IL-12p40 in the CSF correlate with faster conversion to mild cognitive impairment or AD, and predict faster conversion especially for women with elevated TNF-α and all apolipoprotein E (APOE ε4) carriers with elevated IL-9 levels (Contreras et al., 2022). In addition, neuroinflammation is closely related to the cognitive decline of AD patients since inflammatory responses may intensify the pathological process of AD by affecting the metabolism of Aβ and tau protein and causing neuronal damage. Besides, may serve as an early biomarker for AD at an early stage, enabling early diagnosis and disease management (Chen and Yu, 2023).

In the pathology of AD, significant changes occur in cGAS–STING axis. Research has found that the expression and activity of molecules related to cGAS–STING axis are increased in the brains of AD patients (Kinney et al., 2018). For example, in animal models of AD and brain tissues of patients, both the protein and mRNA levels of cGAS and STING are upregulated (Yan et al., 2025). A further study indicates that these changes are closely related to the pathological features of AD. Herpesvirus infection can promote tau-related pathological changes by activating the neuroimmune cGAS–STING axis, while inhibition of STING can improve neuronal dysfunction and reduce neuronal loss (Yan et al., 2025). These results suggest that changes in cGAS–STING axis play an important role in the pathological progression of AD. The relationship between neuroinflammation and the progression of AD pathology is closely linked.

The main pathological features of AD include Aβ deposition, hyperphosphorylation of tau protein with neurofibrillary tangle formation, and neuroinflammation (Webers et al., 2020). Neuroinflammation not only is a pathological characteristic of AD, but can also mediate the pathological of Aβ and tau protein modifications and then promote AD progression. Activated microglia release cytokines could result in tau hyperphosphorylation and contribute to the deposition of neurofibrillary tau tangles (Webers et al., 2020). Inflammatory reactions can interfere with metabolism and elimination of Aβ and lead to an increase of brain Aβ deposition. A study has revealed that inhibition of neuroinflammation in AD mouse model results in a decrease of Aβ deposition, and tau hyperphosphorylation, thus improving cognition (Vom Berg et al., 2012). Moreover, neuroinflammation may also cause damages neurons, and synapses, aggravating the pathological injury of AD. For example, inflammatory factors can damage neuron cell membranes and organelles, disrupting the normal function of neurons, and inducing cell death (Gray et al., 2020).

In the brains of individuals diagnosed with AD, neuroinflammation is a prominent feature, and the main origin of neuroinflammation is the secretion of inflammatory mediators and microglial activation triggered by damage. Excessive and persistent inflammation may damage the nervous system or induce the subsequent emission of damage-associated molecular patterns from compromised cellular structures. Subsequently, these molecules may be recognized by pattern recognition receptors (e.g., NOD-like receptors, TLRs, NLRP3 and cGAS) expressed on immune cells, reigniting the inflammatory response (Gao et al., 2023). Microglia accumulate around the Aβ plaques (Chen et al., 2022b). In the cortical regions of mice utilized as models for AD, elevated levels of caspase-1, NLRP3, and NF-κB were observed. Additionally, positive immunoreactivity for glial fibrillary acidic protein (a biomarker for astrocyte reactivity) and Ionized Calcium-Binding Adapter Molecule 1 (a biomarker of microglial activation) was noted (Govindarajulu et al., 2023). cGAS–STING activation mainly takes place in microglia rather than astrocytes, as demonstrated in a study by Wu et al. (2022).

**Figure 3:**
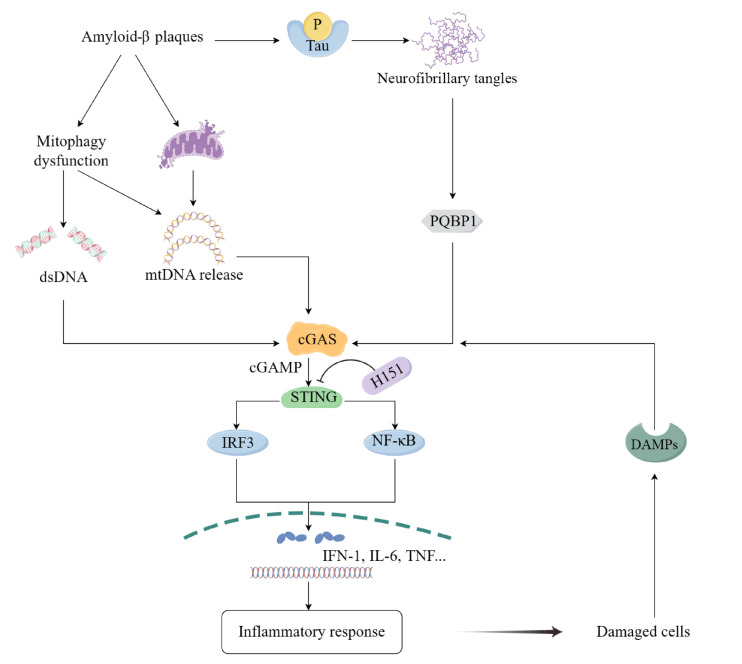
cGAS–STING axis contributes to the pathogenesis of AD. Extracellular accumulation of Aβ leads to impaired mitochondrial autophagy or mitochondrial damage, resulting in the release of mtDNA into the cytoplasm. Concurrently, cellular stress and impaired autophagy contribute to the deposit of cytoplasmic dsDNA, which, together with mtDNA, leads to the initiation of cGAS–STING axis. This cascade not only induces high phosphorylation of tau protein and even NFTs, but also involves PGBP1 acting as an intracellular receptor and activating cGAS–STING axis, which in turn triggers the NF-κB or IRF3 axis, triggering neuroinflammation. AD: Alzheimer’s disease; cGAMP: cyclic guanosine monophosphate-adenosine monophosphate; cGAS: cyclic guanosine monophosphate-adenosine monophosphate synthase; DAMP: damage-associated molecular pattern; dsDNA: double-stranded DNA; H151: a STING antagonist; IFN-I: type I interferon; IL-6: interleukin-6; IRF3: interferon regulatory factor 3; mtDNA: mitochondrial DNA; NFT: neurofibrillary tangle; NF-κB: nuclear factor-κB; PQBP1: polyglutamine-binding protein 1; STING: stimulator of interferon genes; TNF: tumor necrosis factor.

Jin et al. (2021) demonstrated that tau interacts with polyglutamine-binding protein 1 (PQBP1) to facilitate cGAS–STING axis in microglia, promoting pathological progression and ultimately leading to the induction of neuroinflammatory responses, thereby exacerbating AD. In addition, by knocking out the PQBP1 gene, it can be inferred that PQPB1 is instrumental in the tau recognition, leading to the nuclear translocation of downstream key transcription factors IRF3 and NF-κB in cGAS–STING axis. This process ultimately enhanced the gene transcription related to inflammation, such as interferon-stimulated gene 54 (Isg54) and Tnf, suggesting that PQBP1 is involved in the modulation of cGAS–STING axis. The results after extracellular tau monomer injection also revealed that PQBP1 knockout mice showed less cognitive impairment, indicating that PQBP1 may serve as a promising therapeutic target in AD (Jin et al., 2021; **Figure 3**).

Studies on neuroinflammation offer hope in that they might greatly advance our understanding of AD but are fraught with difficulties. Subsequent studies might further clarify the details of mechanisms underlying neuroinflammation in the different AD stages, particularly the relationships between neuroinflammation and pathological changes in Aβ and tau proteins, so that more accurate therapeutic targets could be unveiled. Moreover, it is important to develop better strategies for the control of neuroinflammation, such as searching for new highly specific and sensitive biomarkers and improved imaging strategies to enable dynamic and non-invasive follow-up of neuroinflammatory processes, and optimization of the treatment strategy toward the control of neuroinflammation, in order to enhance the efficiency of drug targeting and safety, and to minimize the side effects. Understanding the inflammatory mechanism of the neuroinflammatory disorder is complicated by the intersection of cell types and signaling molecules involved.

In summary, those studies comprehensively interpret the molecular mechanisms of AD neuroinflammation initiation and progression, describing relations among Aβ deposition, tau protein, cGAS–STING axis and neuroinflammation, to facilitate the integrated comprehension of pathological core of AD. Meanwhile, the discovery of potential drug targets, for example, PQBP1 or verification of the anti-inflammatory mechanism of STING inhibitors provide a theoretical basis for clinical targeted pharmacologic intervention and therapeutic strategies, which leads from bench to bedside, giving the patients hope for future treatment.

Based on the systematic study of signaling pathways, such as cGAS–STING, new therapeutic ideas, which can inhibit disease progression or even reverse pathology so as to alleviate incidences and the rates of disability of AD could be generated in the future. Furthermore, the development of studies on neuroinflammation mechanism of AD will provide lessons of benefit to study other neurodegenerative diseases, giving rise to the progress of neuroscience and providing a fundamental basis for the solution of neurodegenerative disease problems, which effects may bring a change of medical landscape to bring the rise of a better living condition for aging populations and reduces impacts on social welfare healthcare.

### Parkinson’s disease

cGAS–STING axis is also integral to neurological inflammatory diseases, particularly PD (Kwon et al., 2021). The onset of PD is associated with a decrease in dopamine signals in the brain and primarily manifested by bradykinesia, resting tremor, muscle tone, postural and gait abnormalities, and non-motor symptoms, including depression, cognitive decline, decreased sense of smell, fatigue, and apathy (Meoni et al., 2020). There is evidence that inflammation may exacerbate the progression of PD. The neuropathological characteristics of PD mainly consist of the progressive loss of dopaminergic neurons located in the substantia nigra compacta, the proliferation of neural glial cells, and the formation of eosinophilic inclusion bodies, known as Lewy bodies, which primarily contain α-synuclein proteins, within the surviving dopaminergic neurons (Hinkle et al., 2022). Jiang et al. (2023) have revealed that α-synuclein aggregates activate cGAS–STING axis in microglia and astrocytes by inducing mtDNA damage and cytoplasmic release. This axis activates IFN-I response (such as IFN-β) and NLRP3 inflammasome, further exacerbating neuroinflammation and neuronal loss.

Mechanistically, α-synuclein preformed fibers induce DNA double strand breaks γ-H2AX (gamma-H2AX) foci (γH2AX foci) and STING-dependent phosphorylation of TBK1. STING knockout mice showed reduced neuroinflammation, preservation of dopaminergic neurons, and improved motor function in PD models (Hinkle et al., 2022). In addition, the aging of astrocytes driven by cGAS–STING-Yin Yang 1-lipocalin-2 (YY1-LCN2) axis also promotes the progression of PD (Jiang et al., 2023). Aging astrocytes exhibit upregulation of cGAS/STING, impaired mitochondrial function, and secretion of pro-inflammatory factors such as IL-6 and matrix metalloproteinase-3 (MMP-3), which promote dopaminergic neuron death. Hereditary removal of cGAS from astrocytes or drug inhibition of STING (such as H-151) can restore neurodegenerative lesions in 1-methyl-4-phenyl-1,2,3,6-tetrahydropyridine treated mice (Jiang et al., 2023).

**Figure 4:**
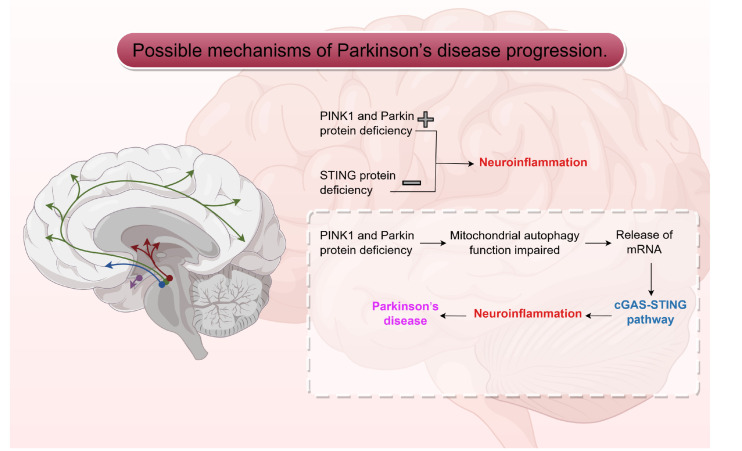
The mechanism of the cGAS–STING axis in neurodegeneration of Parkinson’s disease. Malfunctioning PINK1 and Parkin proteins disrupt the mitophagy process, resulting in the initiation of mtDNA and subsequent secretion of cGAS–STING axis. Moreover, the loss of STING protein can reduce these inflammatory responses. Red arrow: The core region of dopaminergic neuron degeneration in the substantia nigra. Green arrow: Points to the basal ganglia, which receives dopamine input from the substantia nigra. Dysfunction in this area can lead to abnormal motor control. Blue arrow: Other affected brain regions (cortical or brainstem areas involved in later stages), associated with late-stage non-motor symptoms (cognitive impairment and sleep disorders). Black arrow: The main pathway of the core pathological mechanism. cGAS: Cyclic guanosine monophosphate-adenosine monophosphate synthase; mtDNA: mitochondrial DNA; PINK1: PTEN induced putative kinase 1; STING: stimulator of interferon genes.

cGAS–STING axis regulates the polarization of microglia. In PD patients, STING activation is associated with the dominant role of pro-inflammatory type microglia (CD16^+^/CD32^+^), while IRF7 gene knockout shifts microglia towards neuroprotective immunosuppressive type arginase 1^+^/chitinase-like 3^+^ (ARG1^+^/YM1^+^), reducing the expression of TNF-α and inducible nitric oxide synthase (iNOS) (Zhou et al., 2025). Furthermore, positron emission tomography (PET) imaging studies confirm elevated neuroinflammation markers translocator protein (TSPO) in PD patient brains, particularly in the substantia nigra and striatum, correlating with disease severity (Zhang and Gao, 2022; **Figure 4**).

In a word, the cGAS–STING axis is an essential factor in the neuroinflammation of PD. The aggregation of α-synuclein may elicit this signaling pathway and an IFN-I response to activate the NLRP3 inflammasome which enhances neuroinflammation and neuronal damage. This signal at the same time promotes a senescence condition in astrocytes characterized by pro-inflammatory molecule release that stimulates more rapid degeneration of dopaminergic neurons, and regulates the microglial polarization that adjusts the disease course. Knockout and chemical inhibition of the cGAS–STING axis has been also demonstrated to strongly reduce neuroinflammation, preserve the neurons and restore the motor behavior. Our findings in the cGAS–STING axis can inspire future research to revolutionize PD treatments where the cGAS–STING axis might be specifically targeted to slow or even treat PD. These data could also prove useful to understand other neurodegenerative diseases to reach a better understanding of neuroinflammation in terms of general theory but also of neuroscience as a whole field.

### Huntington’s disease

Huntington’s disease (HD) is a genetic disorder that is inherited in an autosomal dominant manner, which has a mean onset age of 35–44 years. It is characterized by a range of motor, behavioral, cognitive, and neuropsychiatric symptoms in a clinical setting (Ghosh and Tabrizi, 2018; Tabrizi et al., 2019). HD pathology arises from the generalized atrophy of the brain and the degeneration of the striatum (caudate and shell nuclei), which is accompanied by the loss of specific medium-sized multis pinning neurons (Gatto et al., 2020), microglial activation, and immune system dysregulation (Sun et al., 2024a). Recent studies have shown that mutant Huntington protein (mHTT) induces DNA double-strand breaks and damages DNA repair by disrupting the interaction between Huntingtin protein (HTT) and MutL homolog alpha (MutLα) (MutL homolog1-postmeiotic segregation increased 2 complex (MLH1-PMS2) complex), leading to MLH1 degradation and overactive DNA cleavage. This process triggers cytoplasmic DNA accumulation, cGAS–STING activation, and subsequent apoptosis in striatal neurons. The autopsy brain of HD showed upregulation of cGAS expression in the striatum and elevated levels of STING-IRF3 phosphorylation, which is associated with upregulation of IFN-stimulated genes and neuronal loss (Sun et al., 2024a).

Mechanistically, mHTT promotes oxidative damage to mtDNA through damaged mitochondrial autophagy, leading to the release of voltage-dependent anion channel 1 (VDAC1) oligomer-dependent mtDNA into the cytoplasm. mtDNA in the cytoplasm activates cGAS–STING axis, driving the production of IFN-I (IFN-α/β) and activating NLRP3 inflammasomes in microglia (Li et al., 2024c). Knocking down of cGAS in HD mouse models (such as R6/2 mice) can reduce IFN-β levels by 60% and restore synaptic plasticity defects, while inhibiting STING can reduce striatal neurodegeneration by 45% (Sun et al., 2024a).

Thus, cGAS–STING axis is significantly linked the inflammatory processes that contribute to the pathogenesis of HD, meaning that the inhibition of this axis could potentially lead to therapeutic interventions for attenuating inflammation-related damage in HD.

### Multiple sclerosis

Multiple sclerosis (MS) is a persistent inflammatory demyelinating disease of the CNS, distinguished by the presence of inflamed regions and the degeneration of neuronal axons, myelin sheaths, and myelin-producing oligodendrocytes (You et al., 2024). This neurodegenerative change is driven by immune dysfunction and subsequent infiltration of immune cells, especially peripheral T cells, B cells, and macrophages, which trigger neuroinflammation and BBB disruption (Vasileiou and Fitzgerald, 2023). In MS, tissue damage is limited to the CNS, while the peripheral nervous system is unaffected (Hauser and Cree, 2020). The initial approval of recombinant IFN-β marked a significant milestone in the pharmacological management of MS. It functions by reducing macrophage and microglial recruitment and myelin phagocytosis. Crucially, the intramuscular injection of IFN-β1 reduces the number and volume of brain lesions in patients with relapsing and remitting MS (Fryer et al., 2021). Furthermore, IFN-α and IFN-β are efficacious treatments in decreasing the frequency of disease recrudescence and restricting lymphocyte soaking in the brain (Wingerchuk and Carter, 2014). By serving as a mediator in the upstream regulation of IFN-β1 production, STING also has a positive role in the treatment of MS. Mathur et al. (2017) showed that the antiviral agent ganciclovir, approved by the U.S. Food and Drug Administration (FDA), was found to elicit IFN-I responses and decrease neuroinflammation through a STING-dependent mechanism, as evidenced by the lack of therapeutic effects in mice lacking the STING protein. In addition, Masanneck et al. (2020) found that the expression of both the GAS and STING genes was lower in patients with relapsed MS than in either patients in remission or healthy individuals. This discovery provides additional evidence supporting the neuroprotective function of cGAS–STING axis in MS and underscores its potential as a target for medical intervention.

Recently, it has been clarified that cGAS–STING axis represents a highly complex and stage specific immune pathway in MS (Johnson et al., 2021; Ferecskó et al., 2023). Activation of STING seems to confer a protective effect on the immune system, mediated through a reduction of effector T cell responses during acute phase of disease. However, several contradictory studies reporting stimulation of STING and significant increase in the concentration and activity of IL-6 secreted by CNS-derived macrophages in MS have been reported (Mittal et al., 2020; Woo et al., 2024). Notably, investigations utilizing the experimental autoimmune encephalomyelitis model have highlighted that, in certain instances, STING activation may not yield therapeutic benefits and could potentially exacerbate neuroinflammation. This complexity underscores the notion that, although STING represents a potential therapeutic target, its effects are highly context-dependent. Hence, a more sophisticated comprehension of its dual function throughout the different states of the illness is very important to timely achieve efficacious treatments.

### Herpes simplex encephalitis

**Figure 5:**
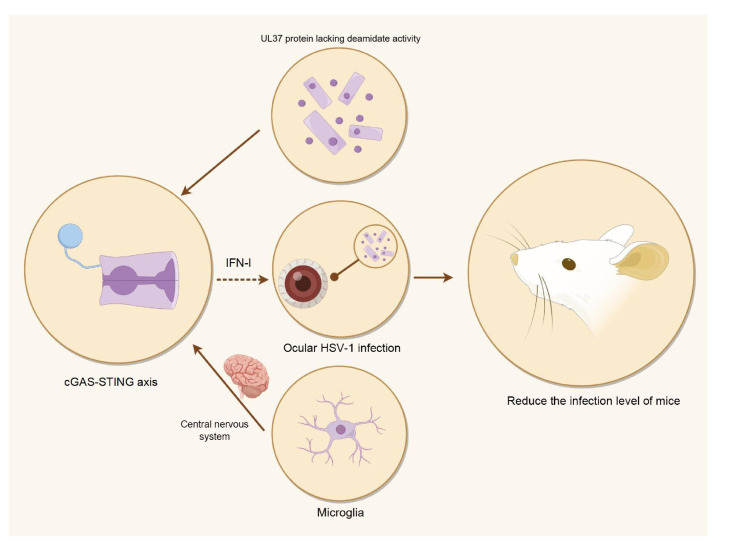
Role of cGAS–STING axis on HSV-1 infection. Following ocular HSV-1 infection, microglia orchestrate the IFN-I response within the CNS through cGAS–STING axis to counteract HSV-1 infection. In addition, when the UL37 protein in HSV-1 mutates and loses its deamidase activity, it also weakens the pathogenicity of the virus in a cGAS- and STING-dependent manner in mice, thereby reducing the degree of HSV-1 infection. cGAS: Cyclic guanosine monophosphate-adenosine monophosphate synthase; CNS: central nervous system; HSV-1: herpes simplex virus-1; IFN-I: type I interferon; STING: stimulator of interferon genes; UL37: unique long 37.

Herpes simplex encephalitis is a prevalent type of sporadic viral cephalitis (Esposito et al., 2022). Herpes simplex virus (HSV), the causative agent of herpes simplex encephalitis, is transmitted through close contact and is neurophilic. People typically become infected with HSV-1 via contact with the mouth and mucous membranes of an HSV-1-positive individual, whereas HSV-2 infection is usually transmitted through sexual intercourse (Rathbun and Szpara, 2021). An innate immune response to IFN-I is essential for resistance to the virus, as it can limit the spread of the virus in neural tissues and reduce disease severity. Building on previous studies that found cytoplasmic viral DNA accumulated in HSV-1-infected macrophages, Reinert et al. (2016) illustrated that microglia have the capacity to coordinate an IFN-I response in the CNS through cGAS–STING axis in order to combat HSV-1 infection. They showed that when mice were deficient in cGAS and STING, they exhibited more severe neurologic symptoms, higher viral loads, and decreased survival rates after ocular infection with HSV-1. Zhang et al. (2018) genetically inhibited UL37 deamidase (an HSV-1 enzyme) to enhance the cytokine response in HSV-1-infected murine models, lower the viral titer in the brain, and promote survival. Typically, unique long 37 (UL37) binds to and deamidates cGAS to decrease its cGAMP synthase activity, thereby reducing IFN-I production and HSV-1 infection clearance. However, a single amino acid variant of cGAS detected in some nonhuman primates increases host antiviral potency against HSV-1. By having an arginine or histidine instead of asparagine at residue position 210, this cGAS variant cannot be deaminated by UL37. Consequently, the animals harboring the cGAS variant mounted strong immunity against HSV-1 (**Figure 5**).

### Ataxia telangiectasia

Ataxia telangiectasia (AT) is a multisystem disorder resulting from mutations in the AT mutated protein (*ATM*) gene, which manifests itself as ataxia and immunodeficiency. Recent studies have revealed that ATM protein kinase, as a guardian of the genome, has functional defects that not only lead to DNA double-strand break repair disorders, but also establish a vicious cycle of neuroinflammation and degeneration by activating cGAS–STING axis (Hu et al., 2021a; Lee and Paull, 2021). In AT patients and animal models, the abnormal accumulation of cytoplasmic DNA (especially mtDNA) caused by ATM deficiency constitutes a sustained activation signal of cGAS–STING axis. This process drives abnormal activation of microglia and secretion of pro-inflammatory factors such as IL-1β and C–C motif chemokine ligand 5 (CCL5), while inducing the senescence-associated secretory phenotype of astrocytes, ultimately forming a neuroinflammatory microenvironment (Yang et al., 2021; Lai et al., 2024). It is worth noting that Talbot et al. (2024) demonstrated through gene editing techniques that inhibiting STING can significantly weaken the chemotactic migration ability of ATM-deficient microglia, suggesting that this axis may participate in neuronal damage by altering the spatial distribution of glial cells.

Mitochondrial homeostasis imbalance is an important amplifier in the pathological process of AT. ATM deficiency leads to mitochondrial autophagy dysfunction, causing damaged mitochondria to accumulate and release mtDNA into the cytoplasm, activating IFN-I response through cGAS–STING axis. A preclinical study has shown that enhancing mitochondrial autophagy through nicotinamide adenine dinucleotide (NAD+) supplementation can effectively block this axis and improve the neurodegenerative phenotype of mouse models (Aguado et al., 2021). It provides a theoretical basis for developing therapeutic strategies targeting the metabolic inflammatory axis.

The limitation of current research lies in insufficient attention to the adaptive immune system. Although AT patients have significant T/B lymphocyte defects, the regulatory mechanism of cGAS–STING axis in these cells and its association with immune failure have not been elucidated (Zaki-Dizaji et al., 2018). In addition, STING activation has a pro-apoptotic effect in lymphocytes, and it is worth exploring whether this characteristic is involved in the formation of AT-specific immune deficiencies (Gulen et al., 2017).

**Figure 6:**
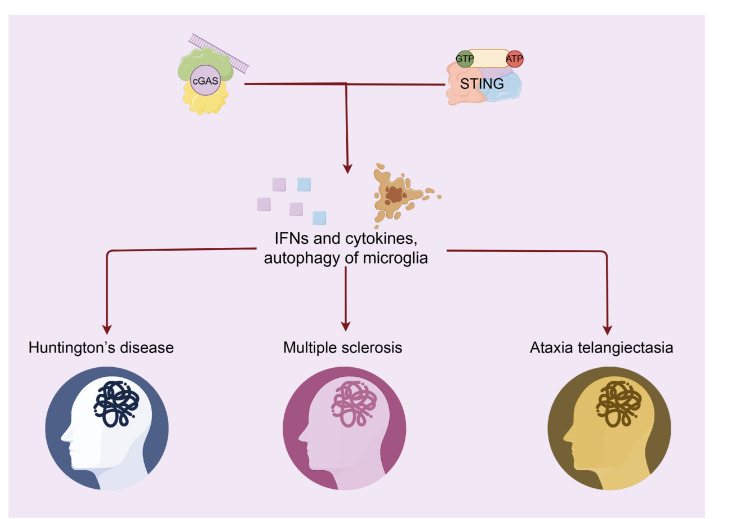
The role of the cGAS–STING axis in the pathogenesis of neuroinflammation. cGAS–STING axis, which is expressed in microglia, significantly influences the pathogenesis of associated neurological diseases. It occurs through the activation of the inflammatory response involving IFN and various cytokines, as well as the promotion of autophagy within microglial cells. ATP: Adenosine triphosphate; cGAS: cyclic guanosine monophosphate-adenosine monophosphate synthase; GTP: guanosine triphosphate; IFN: interferon; STING: stimulator of interferon genes.

Future research needs to focus on addressing the following key issues: (1) whether there is spatiotemporal specificity in the activation of cGAS–STING axis in neurons and non-immune glial cells; (2) How to balance the dual effects of STING inhibition on neuroprotection and immune suppression; (3) The molecular triggering mechanism of mtDNA release and its intrinsic association with ATM function. The answers to these questions will drive a paradigm shift in AT treatment from symptom management to etiological treatment (**Figure 6**).

## Targeting cGAS–STING Axis in the Treatment of Neuroinflammatory Diseases

### Targeted therapy: inhibitors of cGAS–STING axis

A comprehensive examination of the involvement of cGAS–STING axis in the neuroinflammatory response offers a scholarly foundation for the advancement of innovative neuroprotective therapeutic approaches. In particular, the regulation of cGAS enzyme activity has been shown to be critical for neuronal cell protection. In this context, the investigation of inhibitors and activators that modulate the function of cGAS–STING axis has become a focus of therapeutic research.

The Akt kinase can effectively inhibit the activity of the enzyme cGAS by phosphorylating the Ser291 and Ser305 sites (Seo et al., 2015), which provides novel strategies for curbing pathological inflammation. Furthermore, newly developed small molecule inhibitors like RU.320521 or G150 could bind to the enzymatic pocket of species-specific cGAS, thereby effectively inhibiting the composite of cGAMP (Vincent et al., 2017; Lama et al., 2019).

Gamdzyk et al. (2020) have substantiated the correlation between the triggering of cGAS–STING axis and neuronal apoptosis in a neonatal rat model of hypoxic-ischemic injury. They found that lysosome-induced cell death could be slowed down by silencing the *STING* gene or using the cGAS inhibitor RU.320521. However, this protective effect was counteracted by the STING agonist 2′,3′-cGAMP. The cGAS-catalyzed generation of asymmetrically bound 2′,3′-cGAMP is a key aspect of this biological process because it has the highest affinity for STING and facilitates STING dimerization (Gao et al., 2013; Zhang et al., 2013). In addition, it has been hypothesized that LINE-1, as an upstream activator of cGAS–STING axis, was activated after hypoxic-ischemic) events. Moreover, it was revealed that the nucleoside reverse transcriptase inhibitor analog stavudine reduced neuronal cell mortality after hypoxic-ischemia by inhibiting long interspersed nuclear element-1 (LINE-1) (Gamdzyk et al., 2020).

The above findings led to the conclusion that RU.320521, as a cGAS inhibitor, could effectively reduce neurological inflammatory injury, decrease the expression of cathepsin B protein, and inhibit apoptosis. Meanwhile, stavudine was able to slow down degenerative neuronal changes in the cerebral cortex and reduce the extent of apoptosis by downregulating the expression of STING (Gamdzyk et al., 2020). These findings suggest that modulating the activity of cGAS–STING axis may not only alleviate brain injury after hypoxic-ischemia but may also be applicable to the treatment of other related diseases.

cGAS–STING axis is also a key modulator of neuroinflammatory. To protect neurons from excessive inflammation, it is particularly important to develop therapeutic strategies that can precisely control its activity. In pursuit of this objective, a collection of small molecule inhibitors directed towards cGAS–STING axis have been formulated and assessed. For example, anti-malarial drugs such as hydroxychloroquine, inhibit cGAMP synthesis by binding to intracellular DNA, thereby indirectly breaking the interplay between DNA and cGAS (An et al., 2017). Hall et al. (2017) found that PF-06928215, a biologically active small molecule, directly binds to cGAS, combining with its active sites to effectively inhibit its enzymatic activity. This compound has a negligible effect on cellular activity. The new compound “X6” designed by An et al. (2017) mimics the action mechanism of anti-malarial pharmacon and also prevents the binding of DNA to cGAS, albeit with more potency than hydroxychloroquine. Recent studies have shown that suramin, a WHO-recommended essential drug for the treatment of river blindness and African sleeping sickness, also acted as a cGAS inhibitor. Its unique mechanism of action dissociates dsDNA from cGAS, reduces cGAMP production, inhibits cGAS–STING axis, and reduces IFN-I synthesis (Wang et al., 2018).

Glycyrrhiza polysaccharides, licorice flavonoids, and licochalcone B (LicoB), as active components of licorice (Cheng et al., 2021) exert multi-dimensional regulatory effects in inflammatory diseases by targeting cGAS–STING axis (Wen et al., 2023a; Hui et al., 2024; Luo et al., 2024). Moreover, they have all been demonstrated to significantly inhibit cGAS–STING axis and its mediated inflammatory diseases (Wen et al., 2023a, b; Luo et al., 2024). Glycyrrhiza polysaccharide significantly ameliorates systemic inflammation and multi-organ dysfunction caused by intestinal microbiota translocation in the cecal ligation and puncture sepsis model by blocking the binding of STING to TBK1, thereby inhibiting downstream IRF3 phosphorylation and the production of IFN-I (such as IFN-β) and inflammatory cytokines (such as IL-6, TNF-α). This highlights its regulatory capability in host-microbial metabolism interactions (Hui et al., 2024). Licorice flavonoids specifically inhibit cGAMP synthesis, block the activation of cGAS–STING axis, alleviate alveolar damage and inflammatory infiltration in the lipopolysaccharide-induced acute lung injury model, and simultaneously cross-inhibit the NF-κB signaling, reducing the levels of IL-6 and TNF-α in lung tissue, demonstrating the multi-target synergistic advantages of phytochemicals (Wen et al., 2023a). LicoB directly binds to IRF3 and disrupts the formation of the STING-TBK1 complex, thereby inhibiting abnormal activation of IFN-I in the three prime repair exonuclease 1 knockout (Trex1^–/–^) autoimmune model, alleviating splenomegaly and organ inflammation. LicoB, by activating the nuclear factor E2-related factor 2 antioxidant axis, further synergistically inhibits the NLRP3 inflammasome, demonstrating the multi-mechanistic integration characteristic of natural ingredients (Li et al., 2022a; Luo et al., 2024).

The findings showed that Glycyrrhiza polysaccharides, licorice flavonoids, and LicoB exert their anti-inflammatory and pro-immune homeostatic effects by exerting different effects on the upstream, midstream and downstream levels of cGAS–STING axis, and therefore, block the immune homeostasis at a specific node along the signal transduction process. Because they are naturally originated compounds, they have low toxicity and can multi-target actions, thus, making them potential targets for future clinical applications. The data in this report not only might lead to new therapeutic approaches for, among others, sepsis, acute lung injury and autoimmune diseases, but also pave the way to synthesize inhibitors based on the phytochemical structures, for instance, IRF3 binding domain of LicoB. The work puts forward the translational potential of natural medicines towards tailored immune modulation.

As mentioned earlier, cGAS–STING axis is implicated in the development of PD. The emergence of SGK1 inhibitors has found a new strategy for the therapy of PD and related neurological disorders by inhibiting cGAS–STING axis in glial cells to reduce inflammation gene expression. In addition, SGK1 inhibitors reduced the hydrogen peroxide (H_2_O_2_)- and lipopolysaccharide-induced activation of key cGAS–STING axis proteins and decreased the axis expression end product, Ifnb1 mRNA (Kwon et al., 2021). This further confirms the anti-inflammatory properties of serum/glucocorticoid-regulated kinase 1 (SGK1) inhibitors on the NF-κB axis, NLRP3-expressing inflammatory vesicles, and cGAS–STING signaling, thereby affirming their potential efficacy in the therapeutic management of neurological disorders (**Table 1**).

**Table 1:** Agonists and inhibitors for cGAS–STING axis

Drug	Role	Mechanism
Akt kinase	cGAS inhibitor	Effectively inhibits the enzymatic activity of cGAS through phosphorylation at the Ser291 and Ser305 sites
RU320521, G150	cGAS inhibitor	Occupies the enzymatic pocket of species-specific cGAS, thereby inhibiting cGAMP synthesis
Stavudine	cGAS–STING indirect inhibitor	Inhibition of upstream factor LINE-1 activity reduces activation of cGAS–STING axis
Hydroxychloroquine, X6	cGAS inhibitor	Inhibits cGAMP synthesis by binding to intracellular DNA and indirectly blocks the interaction between DNA and cGAS
PF-06928215	cGAS inhibitor	Directly binds to the active site of cGAS and effectively inhibits its enzymatic activity (Hall et al., 2017)
Suramin	cGAS inhibitor	Dissociates double-stranded DNA from cGAS, reduces cGAMP production, and inhibits activation of cGAS–STING axis
SGK1	cGAS–STING inhibitor	Reduces H_2_O_2_- and LPS-induced activation of key cGAS–STING proteins and decreases expression of the pathway end product Ifnb1 mRNA

Akt: Protein kinase B; cGAS: cyclic guanosine monophosphate-adenosine monophosphate synthase; G150: a small molecule inhibitor; H_2_O_2_: hydrogen peroxide; LINE-1: an upstream activator of cGAS–STING axis; LPS: lipopolysaccharide; PF-06928215: a biologically active small molecule; RU.320521: a small molecule inhibitor; SGK1: serum/glucocorticoid-regulated kinase 1; STING: stimulator of interferon genes.

### Clinical research challenges and limitations

cGAS–STING axis has been implicated in neuroinflammatory disorders, including PD and AT, have emerged as a significant area of clinical research (Ding et al., 2022). Aberrant actuation of this axis is strongly related to the development of neuroinflammation, and thus modulation of cGAS–STING axis is suggested as a novel strategy for controlling neuroinflammation and treating related diseases. Clinical studies have begun to explore the use of STING inhibitors to block cGAS–STING axis with a view to reduce nerve cell damage and alleviate disease symptoms (Aguado et al., 2021).

Similarly, cGAS–STING axis is more and more considered to be the therapeutic target in neuroinflammation disease of MS and AD by researchers. For example, an agonist of the STING pathway, cGAMP, shows effective neuroinflammation reduction in the early stage (Xu et al., 2019; Yang et al., 2022). However, limited by the current clinical studies on the cGAS–STING axis, some problems and limitations exist.

First, the cGAS–STING axis might have different functions in multiple diseases and stages of inflammation (Yang et al., 2022), making specific modulation of therapeutic strategies in multiple settings a necessity. Second, clinically relevant biomarkers to assess the rate of disease development and to measure the activity of the cGAS–STING axis have not yet been established, making it difficult to design clinical trials and to interpret their outcome. Moreover, recently it was questioned, also in terms of non-canonical elements of cGAS–STING axis, with consequences possibly far-reaching for the development of the new drugs, and subsequently their clinical relevance.

Ongoing clinical trials and preclinical investigations are examining the specific function of cGAS–STING axis within the CNS to elucidate its potential therapeutic applications for neurological disorders. Enhancing our comprehension of this axis together with the formulation of new strategies for its modulation are expected to offer patients more effective treatment options in the future. However, numerous scientific and clinical challenges need to be overcome before this goal becomes reality.

## Preliminary Clinical Studies and Limitations of Targeting cGAS–STING Axis for the Treatment of Neuroinflammatory Diseases

In the nervous system, the abnormal activation of cGAS–STING axis is closely related to the occurrence and development of various neurodegenerative diseases, such as AD, PD, HD, and MS. Inflammation is a prominent feature of several neurodegenerative diseases, and recent studies have linked the activation of cGAS–STING axis to the development of some of these neurodegenerative lesions (Decout et al., 2021). As the important role of this axis is revealed, many researchers have begun to explore targeting this axis to treat neuroinflammatory diseases. Although targeting cGAS–STING axis for the treatment of neuroinflammatory diseases is considered highly promising and has made some progress, there are still significant challenges in its translational application Particularly concerning the permeability of the BBB and off-target effects. Currently, our researchers are promoting the clinical translation of precision immunomodulatory therapies by optimizing dynamic conformation analysis and targeted delivery systems.

### Preliminary clinical progress targeting cGAS–STING axis

#### Development of small molecule inhibitor

Currently, various small molecule inhibitors targeting cGAS–STING axis have been developed and applied in the treatment of neuroinflammation. These findings provide a theoretical basis for therapeutic strategies targeting cGAS–STING axis inhibitors, laying the foundation for the development of targeted drugs.

*Catalytic site inhibitors*

cGAS antagonists competitively bind to cGAS, which can interfere with the initial activation steps of cGAS. Hall et al. (2017) discovered the pyridazine derivative PF-06928125. They successfully prepared the highly specific anti-cGAMP monoclonal antibody 80-2 by immunizing A/J and SJL/J mice. This antibody can specifically recognize cGAMP without cross-reacting with adenosine triphosphate, guanosine triphosphate or other similar molecules. Based on this, a fluorescence polarization detection method was developed, providing a critical tool for high-throughput screening of cGAS–STING axis inhibitors and research on autoimmune disease treatments. However, its cellular activity is relatively weak, which may be related to competition with high concentrations of nucleotides within the cells. Further structural modifications need to reduce the polar surface area and optimize hydrogen bond donors to enhance permeability.

Vincent et al. (2017) discovered the active site inhibitor RU.521. The study revealed that in bone marrow-derived macrophages from the Trex1^–/–^ autoimmune mouse model, the small molecule inhibitor RU.521 significantly reduced the constitutive overexpression of IFN-β1 mediated by cGAS–STING axis by specifically inhibiting the enzymatic activity of cGAS. It showed no significant impact on other immune pathways, demonstrating its effective targeted regulation of cGAS–STING axis. In mouse macrophage cell line RAW264.7, the inhibition of dsDNA-induced cGAS signaling selectively suppresses Ifn-b1 mRNA expression in Trex1^–/–^ mouse bone marrow-derived macrophages without affecting other innate immune axes. Lama et al. (2019) discovered the pyridine and indole tricyclic core derivative G150. The study, utilizing gene knockout mouse models such as TREX1-deficient mice, discovered that inhibiting cGAS could block autoimmune responses and rescue lethal phenotypes, thereby confirming the pivotal role of cGAS–STING axis in inflammatory diseases triggered by aberrant DNA. However, the primary drug experiments were predominantly conducted on human primary macrophages and cell models, without extending to *in vivo* drug efficacy validation. It has no off-target effects in THP-1 cells and primary macrophages. Zhao et al. (2020) identified S3 through a combination of thermal transition experiments. Its combination mode is similar to PF-06928125 and RU.521, but the differences in activity suggest that virtual screening can complement traditional methods, providing a new starting point for the development of novel inhibitors.

*DNA-binding interference inhibitors*

The derivatives of antimalarial drugs, hydroxychloroquine, quinine, and second-generation molecule X6, were demonstrated to down-regulate IFN-β induction by intercalating into the minor groove of the DNA to hinder the cGAS-dsDNA interaction (An et al., 2015, 2017, 2018). In preclinical experiments, artemisinin derivatives, as well as quinoline/acridine antimalarial drugs, have been shown to block the association between cGAS and DNA in animal models and mice, e.g., MRL/lpr and NZB/W F1 mouse models, thereby inhibiting the cGAS–STING axis and reducing the levels of IFN-I such as IFN-β. Thus, this inhibition alleviates the lupus-like symptoms such as decreases of levels of anti-DNAs and kidney damage improving the survival rate. Moreover, investigations conducted with TREX1-deficient mouse models have shown that loss of cGAS fully prevents disease, highlighting the importance of the cGAS axis in autoimmunity disease pathogenesis. Trex1^–/–^ mouse model Oral administration of X6 significantly reduced the levels of interferon-stimulated genes, within spleen and heart, and it down-regulated cGAMP levels within the heart, via the blockade of cGAS–STING axis. It also supports the strategy of X6 to act as the target and block the pathway that it might also serve as a proof-of-concept therapy for cGAS/STING-driven autoimmune disease. As mentioned above, in mice lacking TREX1, the accumulation of intracellular DNA triggers cGAS–STING axis and IFN-β expression. We have recently identified the antimalarial antivirals including quinacrine as cGAS–STING inhibitor that their mechanism involves these mechanisms and ultimately prevent the expression of IFN. Therefore, we have argued that these molecules may have indirect negative influence on cGAS by forming complex with dsDNA and blocking the interaction of cGAS with dsDNA (Decout et al., 2021).

Wang et al. (2018) identified that sulfamethoxazole can competitively bind to the positively charged region of cGAS, disrupting its interaction with dsDNA. Decout’s animal experiments show that gene knocking or pharmacologically inhibiting cGAS–STING axis (e.g., treating Trex1-deficient mice with the STING inhibitor C-176 or deleting STING in Parkin/Pink1-deficient mouse models) significantly reduces inflammatory cytokine levels, ameliorates neurodegenerative and cardiac inflammatory conditions, and alleviates aging-related phenotypes (Decout et al., 2021). Wang et al. (2018) confirmed the critical driving role of this pathway in various diseases and its potential as a therapeutic target. Although this molecule carries a high charge, it exhibits cellular activity and has no effect on the TLR axis. The inhibitory oligonucleotide A151 suppresses the IFN response in TREX1-deficient cells by specifically binding to the DNA-binding domain of cGAS, providing a strategy for the treatment of DNA-driven autoimmune diseases (Decout et al., 2021).

*Development strategies for STING inhibitors*

The design of STING inhibitors focuses on two types of targets. The first is the cyclic dinucleotides (CDN) binding site antagonists: these mimic CDN occupying the ligand pocket, thereby blocking STING activation. The second is a palmitoylation site inhibitor: targeting Cys88/Cys91, interfering with STING membrane localization and signal transduction (Decout et al., 2021). The animal experiments demonstrated that knocking out genes or pharmacologically inhibiting cGAS–STING axis (e.g., treating Trex1-deficient mice with the STING inhibitor C-176 or deleting STING in Parkin/Pink1-deficient models) significantly reduces inflammatory cytokine levels, ameliorates neurodegenerative and cardiac inflammatory conditions, and alleviates aging-related phenotypes (Decout et al., 2021). These findings confirm the critical driving role of this pathway in various diseases and its potential as a therapeutic target.

*CDN combined with site antagonists*

Research has found that the C-terminal domain of the STING protein exists in a symmetric dimer form, with the ligand binding site located at the interface of the two monomers. Siu et al. (2019) used a symmetric design to create dual molecular antagonists, obtaining a low-affinity compound 1 through mass spectrometry screening. X-ray structure shows that two molecules occupy the binding site and induce STING to adopt an inactive conformation. Based on polarity/hydrophobic interactions and acid pKa optimization, a highly efficient compound 18 was ultimately obtained, which inhibits cAMP-induced IFN-β secretion in THP-1 cells in a bimolecular manner.

Li et al. (2018) screened the natural product astin C from a cyclic peptide library, confirming its competitive binding to the CDN site of STING, blocking the recruitment of IRF3 and inhibiting the signaling axis. The study, utilizing the Trex1^–/–^ mouse model, discovered that the cyclic peptide Astin C significantly inhibits cGAS–STING axis-mediated expression of IFN-I and pro-inflammatory factors by specifically binding to STING and blocking the recruitment of IRF3 to its signalosome. This effectively alleviates autoimmune inflammatory responses and reduces serum autoantibody levels. Their *in vitro* experiments show that this compound can inhibit the expression of IFN-I, and *in vivo* administration in the form of cyclodextrin inclusion significantly reduces the transcription of inflammation-related genes such as *Ifnb* in multiple tissues of mice. The regulatory role of the STING axis has been validated (Li et al., 2018).

*Targeting the palmitoylation sites of STING*

Haag et al. (2018) obtained nitrofurans derivatives C-176/C-178 through cell screening, confirming their specific binding to the Cys91 site of STING, thereby blocking palmitoylation modification and signal axis activation. This study, utilizing the Trex1^–/–^ mouse model, demonstrated that covalent small-molecule inhibitors targeting the Cys91 site of the STING protein (such as C-176 and H-151) effectively inhibit STING palmitoylation and its downstream signaling. This significantly reduces the levels of IFN-I and IL-6, alleviates multi-organ inflammatory pathological features, and provides experimental support for treating autoinflammatory diseases associated with abnormal activation of cGAS–STING axis. *In vitro* and *in vivo* experiments have shown that this compound significantly inhibits the secretion of STING-mediated inflammatory factors. Structural optimization reveals that the nitro and furan groups are essential for activity, and some derivatives exhibit inhibitory activity against both human and mouse STING (Decout et al., 2021).

Haag et al. (2018) obtained the indole derivative H-151 through cell screening, confirming that it inhibits IFN-I response and TBK1 phosphorylation by covalently binding to the Cys91 site of STING. The study effectively suppressed the hyperactivation of cGAS–STING axis in the Trex1^–/–^ mouse model by targeting the Cys91 site of STING using covalent small molecule inhibitors C-176 and H-151. It significantly reduced serum levels of IFN-I and multi-organ inflammatory responses, demonstrating the potential of STING inhibitors in treating autoinflammatory diseases. *In vivo* experiments show that this compound significantly inhibits the systemic inflammatory response induced by STING agonists and reduces IFN-β levels in mouse models (Decout et al., 2021), revealing its mechanism of action by covalently modifying and inactivating STING.

Hansen et al. (2018) discovered that nitro-fatty acids (such as nitro-oleic acid) generated by HSV infection covalently modify Cys88, Cys91, and His16 of STING through Michael addition, inhibiting its mediation of IFN-I responses and significantly reduce the levels of phosphorylated TANK-binding kinase 1 (pTBK1) in STING cells with SAVI mutations. They, utilizing a murine vaginal HSV-2 infection model, discovered that viral infection induces the generation of iNOS-dependent nitro fatty acids nitro-oleic acid. These acids covalently modify the Cys88 and Cys91 sites of the STING protein, inhibiting its palmitoylation and the activation of cGAS–STING axis, thereby significantly reducing the release of IFN-I.

Vinogradova et al. (2020) used chemical proteomics methods to discover that acrylamide active acrylamide (BPK-21/25) binds to Cys91 of STING and other immune proteins’ cysteines Inhibit cAMP-mediated STING activity and propose a binding model. The study did not involve animal experiments, but it revealed that the compound BPK-25, through covalent modification of the C91 cysteine residue on the STING protein in human T cells, inhibits cGAMP-induced STING activation and the production of inflammatory factors (e.g., IFN-β). This discovery elucidates the chemical intervention mechanism targeting cGAS–STING axis.

#### Applications of gene editing technology

Clustered regularly interspaced short palindromic repeats - CRISPR-associated protein 9 (CRISPR-Cas9) technology is one of the most commonly used gene editing techniques, and it also offers new possibilities for targeting cGAS–STING axis. Researchers specifically knocked out the cGAS or STING genes using gene-editing techniques, thereby disrupting the continuity of this pathway and preventing its activation, which also significantly reduced the pathological changes caused by neuroinflammation (Lu et al., 2022). For example, researchers successfully constructed the tumor microenvironment responsive nanoplatform hyaluronic acid-modified manganese dioxide nanoparticles loaded with MSA-2 and plasmid encoding Cas9/sg-PD-L1 (HMnMPH), designed for dual activation of cGAS–STING axis in conjunction with *CRISPR-Cas9* gene editing for cancer immunotherapy. HMnMPH is formed by the HA modification of HMnO_2_, which carries the STING agonist MSA-2 and the Cas9/sg-PD-L1 plasmid. It can suppress primary and distant cancers, promoting the proliferation of memory T cells for long-term tumor elimination. Its mechanism lies in the activation of cGAS–STING axis to release IFN-β, as well as the inhibition of programmed death-ligand 1 (PD-L1) to promote the action of immune cells and stimulate the proliferation of memory T cells (Lu et al., 2022). However, due to the involvement of ethical issues and safety concerns in the clinical application of actual diseases, further optimization is also required.

### Positron emission tomography tracers visualize the activation state of STING

It has been found using PET tracers that the level of STING activation is closely associated with the efficacy of disease treatment (Xu et al., 2023). In the research of neuroinflammatory diseases, PET tracers have also demonstrated great potential. It can help researchers gain a deeper understanding of the activation patterns and dynamic changes of cGAS–STING axis in neuroinflammatory diseases, guiding drug development and optimizing treatment plans. Monitoring the changes in STING activation status through PET imaging can assess the efficacy of drugs targeting cGAS–STING axis and allow for timely adjustments to treatment strategies.

PET technology has been increasingly applied in medical research and clinical diagnosis, holding potential applications (Fang et al., 2024). In recent years, the development of PET tracers capable of visualizing the activation state of STING has become a research hotspot. By designing specific PET tracers, we can monitor the activation status of STING in real-time at the *in vivo* level, providing important imaging evidence for therapies targeting cGAS–STING axis.

STING is an important component of the innate immune system and plays a crucial role in tumor immunotherapy. Developing non-invasive *in vivo* diagnostic methods to observe STING is highly valuable for STING-related immunotherapy (Liu et al., 2025). Researchers utilized the STING-targeted radioactive probe [^18^F]fluoro-STING imaging probe 1 ([^18^F]F-CRI1) to conduct *in vivo* PET imaging on STING-positive tumors (B16F10, MC38, and Panc02) (Liu et al., 2025). By quantitatively analyzing the expression levels of STING protein in tumor tissues and integrating imaging data for a comprehensive analysis, Liu et al. (2025) confirmed that the uptake of [^18^F] F-CRI1 in PET imaging of tumors shows a significant positive correlation with STING expression levels. In recent years, professor Liu’s research team has successfully developed a PET tracer targeting the STING protein, which can accurately identify activated STING molecules and achieve *in vivo* imaging (Liu et al., 2025). Based on the structure of the STING inhibitors C170/C176, researchers have designed and synthesized novel radioactive ligands specific inhibitor-based radioligand [^131^I]Iodo-NFIP (^131^I-NFIP) and specific inhibitor-based radioligand [^18^F]Fluoro-NFEP (^18^F-NFEP) (Fang et al., 2024). Obtain tracers with high purity (> 95%) and high specific activity (28.56–48.89 GBq/μmol) through isotope labeling technology. Cellular experiments confirmed that ^131^I-NFIP has a high affinity, and its tumor uptake is significantly positively correlated with the expression level of STING protein. This study first established a radioactive probe based on STING inhibitors, providing an innovative tool for the non-invasive visualization of the STING activation status in the tumor microenvironment. The research team successfully developed a novel STING-targeted PET tracer specific inhibitor-based radioligand a high affinity STING agonist-benzothiophene derivative[^18^F]Fluoro-FBTA (^18^F-FBTA) (Xu et al., 2023). The labeling process has a high radiochemical yield of 79.7% ± 4.3% and a molar activity of 32.5 ± 2.9 GBq/μmol. Experiments confirmed that the tracer has a high affinity binding characteristic to the STING protein with a value of 26.86 ± 6.79 nM. Early warning of pulmonary inflammation and evaluation of drug efficacy can be achieved through PET imaging in a mouse model of acute lung injury. Compared to traditional specific inhibitor-based radioligand 2-[^18^F]Fluoro-2-deoxy-D-glucose (^18^F-FDG) imaging, ^18^F-FBTA demonstrates higher tissue specificity and distribution advantages, enabling precise tracking of acute lung injury progression before the pathological features are revealed in X-ray computed tomography. This study provides innovative tools for the *in vivo* visualization of STING as an inflammatory biomarker, demonstrating its potential for clinical translation.

### Limitations of targeting cGAS–STING axis in neuroinflammation

Despite the significant potential of targeting cGAS–STING axis in neuroinflammatory diseases, its translation into clinical applications still faces several notable limitations.

#### Permeability of blood–brain barrier

The BBB is a precisely regulated barrier composed of endothelial cells, astrocytes, and pericytes, whose core function is to maintain the stability of the brain’s microenvironment through a selective filtration mechanism. This barrier system selects substances based on characteristics such as molecular size, charge properties, and lipid solubility, effectively preventing the invasion of harmful substances while maintaining the normal supply of nutrients (Zhou et al., 2024). The BBB, as an important component of the body’s natural defense system, effectively protects the CNS through a tightly arranged layer of endothelial cells. However, this strict defense mechanism also poses challenges for drug delivery—many drugs specifically designed for brain diseases often encounter obstacles when crossing this biological barrier, resulting in significantly reduced therapeutic efficacy. Taking small molecule inhibitors targeting cGAS–STING axis as an example, these drugs have shown potential in the treatment of neuroinflammatory diseases, but their efficacy is limited due to difficulties in penetrating the BBB.

Laboratory studies indicate that most drugs can exhibit pharmacological activity in *in vitro* experiments, but once they enter the body, they are unable to reach effective concentrations in brain tissue due to the physiological limitations of the BBB (Martins and Sarmento, 2022; Wang et al., 2022; Al Rihani et al., 2023). In response to this challenge, researchers have attempted to enhance the lipophilicity of drugs through molecular structure optimization, or to utilize the body’s natural transport protein system to carry drug molecules across this physiological barrier (Banks, 2009; Pardridge, 2012). The application of these strategies provides new insights for overcoming the bottleneck of drug delivery across the BBB. In response to the challenges of drug delivery across the BBB, researchers are exploring nanocarrier technology. By encapsulating drugs with materials such as nanoparticles and liposomes, and utilizing targeted delivery and sustained release characteristics to overcome barrier limitations, this approach is particularly suitable for treating neurological diseases with narrow therapeutic windows. Bionic nanotechnology systems and innovative platforms such as smart hydrogels can precisely target neuronal mitochondria and extend the duration of drug action, significantly enhancing the therapeutic effects for conditions such as AD and PD. This type of technology, through the interdisciplinary integration of materials science and neuroscience, not only addresses the traditional delivery bottlenecks but also brings revolutionary breakthroughs to neuroprotection It is expected to expand to more clinical applications for neurodegenerative diseases in the future (Palle and Neerati, 2018; Rajput et al., 2018; Han et al., 2020). Nevertheless, the safety, stability, and long-term effects of nanocarriers remain unknown and require further investigation for validation.

#### Off-target effects and systemic side effects

cGAS–STING axis is widely distributed across various tissues and regions of the body. Targeted therapy for this axis may also trigger off-target effects. The therapeutic potential of targeting cGAS–STING axis is a double-edged sword. Although its excessive activation is associated with disease progression, complete suppression may disrupt the necessary immune response. For example, although STING inhibitors have certain effects on alleviating neuroinflammation and neurodegeneration in some disease contexts and microenvironments, their suppression of immune responses must be considered in the context of risks such as infections and tumors, therefore, the balanced regulation is crucial (Zhang et al., 2024b). Inhibiting the activity of STING is likely to lead to a decline in the overall immune function of the body, increasing the risk of tumors and infections (Zhang et al., 2024b). Currently, most of the targeted drugs developed for the STING axis are non-specific inhibitors, which are likely to have some negative effects on other physiological functions. Therefore, whether it is possible to develop specific targeted therapies for inflammatory diseases and reduce the impact of these therapies on other targeted tissues and systems is the core issue of our current research.

#### Bottlenecks in preclinical research and clinical translation

Currently, therapeutic strategies targeting cGAS–STING axis have achieved significant results, but since ultimately all therapeutic strategies must be applied to humans. How to translate these research findings into clinical treatment is another issue. Considering that neuroinflammatory diseases involve many complex mechanisms, it is necessary to adjust treatment strategies to meet the individualized treatment needs of different patients when facing various individuals. First, the available treatment methods lack sufficient specificity, making it difficult to achieve individualized adjustments. Second, existing preclinical studies mostly focus on acute nerve injuries or short-term immune responses, and there is still a lack of treatment plans targeting chronic long-term neuroinflammation. Finally, the therapeutic targeting of cGAS–STING axis requires a substantial amount of clinical trial data for efficacy evaluation in clinical translation and safety.

### Future development directions

Although the regulatory mechanisms of cGAS–STING axis in neuroinflammation and neurodegenerative diseases are complex, its potential as a therapeutic target is gradually becoming apparent with the deepening of research. The current challenges include technical bottlenecks in precise regulation and difficulties in safety assessment. Although it is a potential therapeutic target, its operation must be approached with caution. Future research should focus on elucidating the molecular mechanisms of cGAS–STING axis, developing targeted drugs in conjunction with novel delivery systems, and validating their efficacy and risks through multidimensional clinical trials. Continuously exploring the dynamic role of this axis in the disease process will provide new strategies for the treatment of neurodegenerative diseases such as AD.

#### Development of precision drug delivery systems

Future research on targeted therapy strategies place greater emphasis on the development of precise drug delivery systems, enhancing the distribution of targeted drugs within the nervous system and ensuring accuracy in targeting specific locations. Through gene editing technology, nanotechnology, and precise drug delivery technology, combined with small molecule drugs, antibodies, and RNA interference technology, researchers can achieve precise regulation of cGAS–STING axis Overcoming the permeability issues of the BBB and the occurrence of off-target effects.

#### Advancement of personalized treatment and clinical trials

With the in-depth study of the genetic mechanisms of neuroinflammatory diseases, precision medicine is gradually realizing the development of personalized treatment plans (Zhou et al., 2024). Although this gene-based customized strategy can optimize efficacy, drug development must conduct treatment validation for different genetic backgrounds, significantly increasing technical complexity.

At the same time, the risks of data privacy breaches and potential genetic discrimination issues have sparked ethical controversies. Despite facing multiple challenges, breakthroughs in neurodegenerative diseases are expected through the deepening of research into disease mechanisms, innovations in delivery technologies, and the establishment of clinical-research collaboration networks. In the future, it is necessary to seek a balance between technological innovation and ethical standards, promoting the translation of precision medicine from the laboratory to clinical practice.

## Future Prospect of cGAS–STING Axis

### Future directions for cGAS–STING axis

#### Deepening of molecular mechanisms

A comprehensive elucidation of the molecular mechanisms governing cGAS–STING signaling represents a critical avenue for future research endeavors. This research is expected to focus on the following key areas:

The precise roles of the cGAS and STING proteins: Researchers will meticulously analyze the specific functions and activities of the cGAS and STING proteins, as well as other proteins implicated in cGAS–STING axis. More information is needed regarding how these proteins recognize DNA, how they localize within the cell, and how they interact with other proteins. The dynamics of structural changes and how they affect signaling at the molecular level should also be explored.

Mechanisms regulating signaling: Researchers investigate the regulatory processes governing cGAS–STING axis as a reaction to varying cellular conditions and external stimuli. This involves studies of how triggers such as DNA damage, pathogenic infection, or cellular stress responses activate the axis. In addition, there is a need to study how cGAS–STING signaling is terminated, and the inhibitory proteins and feedback mechanisms involved in this process.

Axis influences on cellular fate: cGAS–STING axis is integral to the immune response and may also potentially exert significant effects on cellular survival, proliferation, differentiation, and apoptosis. The cGAS–STING axis possesses dual regulatory functions in immune activation and cell death, capable of inducing various forms of death such as apoptosis and pyroptosis, and demonstrates significant potential in the treatment of tumors, infections, and autoimmune diseases. As a critical bridge between innate immunity and cell death, this pathway is involved in processes such as inflammation, metabolism, and genome stability. Research into the mechanisms of the cGAS–STING axis not only advances the development of tumor and antiviral therapies but also provides new insights into autoimmune diseases and Neurodegeneration. Elucidating its interactions with the death network will offer an important theoretical foundation for the study of disease mechanisms and targeted therapies. Thus, future studies can evaluate the role of this axis in determining cell fate and explore how it impacts cellular physiology and pathology.

Studies of non-canonical axis: While cGAS and STING typically function in concert within the cGAS–STING axis, there are documented cases where cGAS operates independently of STING, or STING functions without the involvement of cGAS. This finding not only enhances our comprehension of the cGAS–STING axis but also offers novel insights into its implications for immune responses, inflammatory diseases, and tumorigenesis. As research into the cGAS–STING axis advances, it is becoming increasingly apparent that the regulatory mechanisms governing the non-canonical axis are more intricate than those of the canonical axis, involving a diverse array of cell types and their interactions. Future research should investigate the existence and significance of these non-canonical pathways, as well as their impact on intracellular signaling and cellular responses. This investigation may require examining molecules beyond cGAS and STING and assessing their roles across various cell types and pathophysiological conditions. Furthermore, this approach implies that in the exploration of disease mechanisms, reliance solely on classical immune models is insufficient; instead, integrating non-canonical pathways is essential to achieve a more comprehensive understanding of the dynamic processes underlying immune responses.

Revealing axis diversity and complexity: Different cell types may respond differently to cGAS–STING axis. For example, immune cells and neural cells may respond differently to the same signal because of their different physiological functions. Future studies could reveal this diversity and complexity, as well as explore how cGAS–STING axis is regulated under different physiological and pathological states and how this affects disease outcomes.

Although the central role of cGAS–STING axis in CNS diseases has been confirmed, critical gaps remain in understanding its spatiotemporal regulatory network. Breaking through this bottleneck requires the integration of multimodal technologies with single-cell resolution and temporal tracking capabilities. Chen et al. (2020), utilizing spatial transcriptomics and *in situ* sequencing technologies, identified two major co-expression networks, plaque-induced genes and oligodendrocyte genes, in the brain tissues of APPNL-G-F mice and human AD patients. Plaque-induced genes are involved in the complement cascade, oxidative stress, and immune responses, mediated by microglia and astrocytes, and are enriched near amyloid plaques in AD patients. Oligodendrocyte genes are associated with myelination, activated under mild amyloid stress, and depleted in high-load regions. In the future, the integration of spatial transcriptomics with single-cell technologies, multi-omics, and cross-species models could elucidate the mechanisms of cellular interactions in AD. Plaque-induced genes and oligodendrocyte genes may emerge as novel therapeutic targets, expanding spatial transcriptomics research into pathways such as the cGAS–STING axis, potentially uncovering common mechanisms in diseases like AD.

**Figure 7:**
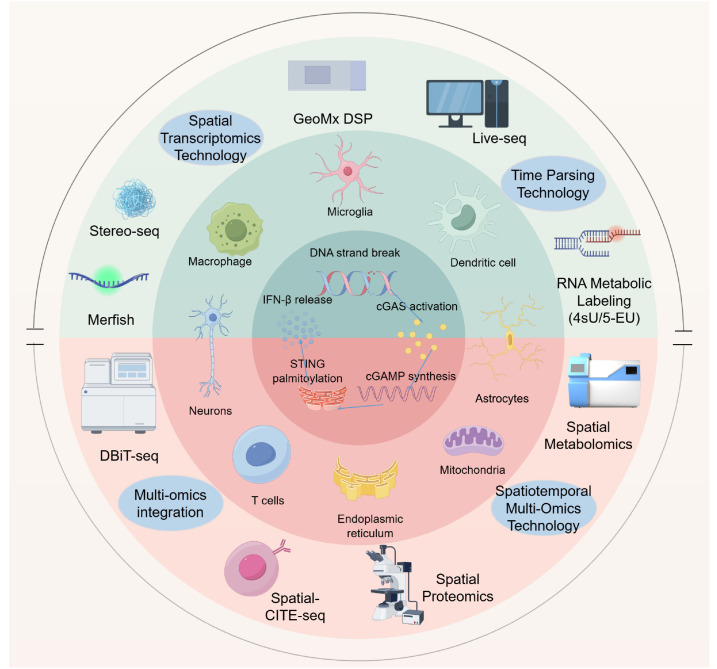
Application of cutting-edge spatial and temporal resolution technology in cGAS–STING axis. Integrating cutting-edge spatial and temporal resolution technologies will enhance the exploration of cGAS–STING axis in central nervous system homeostasis and neuroinflammatory pathogenesis. Center: Flowchart of cGAS–STING axis. Central ring: Major cells and organelles involved in central nervous system homeostasis and neuroinflammatory pathogenesis, such as microglia, neurons, macrophages, T cells, astrocytes, dendritic cells, mitochondria, and endoplasmic reticulum. Outer ring: Technical modules. Top-left quadrant: Spatial transcriptomics technology. Top right quadrant: Time-resolved technology. Lower-left quadrant: Multi-omics integration. Bottom-right quadrant: Spatiotemporal multi-omics technology, spatial dimension. cGAS: Cyclic guanosine monophosphate-adenosine monophosphate synthase; DBiT-seq: deterministic barcoding in tissue for spatial omics sequencing; IFN-β: interferon-β; Stereo-CITE-seq: spatially-resolved transcriptomics and epigenomics by sequencing with co-detection of immunoglobulin and transcriptome expression; Stereo-seq: spatially-resolved transcriptomics and epigenomics by sequencing; STING: stimulator of interferon genes.

Further integration of temporal resolution techniques (e.g., *in vivo* two-photon microscopy tracking in STING-GFP mouse models) can capture dynamic signaling events between astrocytes and infiltrating T cells during pathological progression. The spatiotemporal multi-omics technologies proposed by Chu et al. (2024) provide tools (such as single-cell transcriptomics and spatial proteomics) for deciphering the spatiotemporal activation patterns of the cGAS–STING axis in specific cell subpopulations or tissue regions, particularly in the study of molecular mechanisms underlying aging-related diseases (e.g., neurodegeneration) or regenerative disorders. In the future, by integrating multimodal data and artificial intelligence analysis, the spatiotemporal regulatory networks of the cGAS–STING axis in aging and regeneration can be further explored. However, its specific roles need to be clarified through functional validation experiments, and the current limitations in high-resolution spatiotemporal dynamic tracking must be overcome to achieve breakthroughs from mechanisms to translation. More systematic breakthroughs will come from spatiotemporal multi-omics integration strategies such as synchronously capturing transcriptomic, proteomic, and metabolomic information from the same brain microenvironment via spatially-resolved transcriptomics and epigenomics by sequencing with co-detection of immunoglobulin and transcriptome expression (Stereo-CITE-seq). Combined with pseudotime analysis algorithms, this approach can reconstruct the cascade process mediated by cGAS–STING: mtDNA leakage → innate immune activation → autophagy inhibition. This multi-dimensional data-driven research paradigm not only precisely identifies key nodes in regulatory networks (such as the spatiotemporal specificity of STING palmitoylation modification) but also validates potential intervention targets through spatial CRISPR screening. Ultimately, the establishment of a trinity research system encompassing human brain organoids, live imaging, and clinical samples will provide theoretical support for the development of spatiotemporally selective STING modulators (e.g., BBB-penetrating nano-inhibitors). It will drive a paradigm shift in neuroinflammation treatment from broad-spectrum suppression to precise regulation (**Figure 7**).

#### Interaction with other signaling axes

The interplay between cGAS–STING axis and other axes is anticipated to be a pivotal focus of forthcoming research. Investigations in this domain are expected to enhance our comprehension of the intricate nature of intracellular signaling networks and the multifactorial etiology of neurological disorders. The interactions between cGAS–STING axis and its downstream effects warrant particular academic interest:

Autophagy: Autophagy is the process of cell self-phage and digestion, which helps to remove damaged proteins and organelles, thereby being a key factor in maintaining the homeostasis of the intracellular environment (Lei and Klionsky, 2021). The interaction between cGAS–STING axis and autophagic mechanisms may be a critical mechanism in various diseases, encompassing neuroinflammatory disorders, cancer, and infectious diseases. In the context of neuroinflammatory conditions, the cGAS–STING axis may play a critical role in modulating inflammatory responses and preserving neuronal viability through the regulation of autophagy. To advance our understanding, it is imperative to elucidate the molecular mechanisms by which the cGAS–STING axis influences inflammatory responses via autophagy, with particular emphasis on the precise process through which STING undergoes autophagic degradation to establish a negative feedback loop. Furthermore, research should focus on CNS injuries, where dysfunctional autophagy results in sustained activation of the cGAS–STING axis, thereby intensifying neuroinflammation and promoting neuronal cell death. Finally, investigating novel therapeutic strategies that target the autophagy-STING interaction—such as enhancing autophagy to eliminate cytoplasmic DNA or modulating the lysosomal degradation of STING—may provide promising avenues for neuroprotection. Future research should aim to elucidate the differential regulation of the cGAS–STING axis by autophagy across various cell types, including microglia and neurons, and to explore the therapeutic potential of these mechanisms in both acute and chronic neurological disorders. Such findings will provide a foundational basis for the development of targeted interventions aimed at the cGAS–STING-autophagy axis.

Apoptosis: Apoptosis can affect different cell populations, including neural precursor cells, glial cells, and differentiated neurons. This process ensures the survival of cells that are both morphologically and functionally normal, and, in the case of neurons, effectively integrated with their synapses and axons. Apoptosis is critical for neural function and tissue health; its dysregulation may result in a range of pathologies (Sharma et al., 2021) and neurological inflammatory disorders such as PD (Li et al., 2022b). cGAS–STING axis has the potential to engage with the apoptotic axis via various mechanisms (Zheng et al., 2023). Numerous studies have demonstrated that the initiation of cGAS is initiated when DNA damage within cellular tissues. This in turn activates STING, which regulates the production of inflammatory and chemotactic factors, inducing immune responses, inflammatory reactions, and apoptosis (Yu et al., 2020). Thus, the association between cGAS–STING axis and apoptosis may be pivotal in antiviral responses, tumor immunosurveillance, and certain neurodegenerative diseases.

Crosstalk with other inflammation-associated axes: cGAS–STING axis holds a significant position in the regulation of inflammatory responses; nevertheless, its interplay with other inflammatory axes, such as NF-κB and Janus kinase-signal transducer and activator of transcription (JAK-STAT), is of comparable significance. For example, neuronal senescence after ischemic stroke can be ameliorated by inhibiting cGAS–STING activation by targeting the JAK2-STAT3 axis (Zhang et al., 2023a). Furthermore, severe acute respiratory syndrome coronavirus 2 (SARS-CoV-2) infection has the potential to specifically activate NF-κB signaling through cGAS–STING axis, leading to the generation of numerous pro-inflammatory cytokines. Consequently, targeting cGAS–STING axis may represent a viable therapeutic strategy for the treatment of severe respiratory diseases, including coronavirus disease 2019 (COVID-19) (Neufeldt et al., 2022). Crosstalk between these axes may affect the initiation of immune cells, the secretion of inflammatory factors, and the persistence of the inflammatory process. In neuroinflammatory diseases, autoimmune diseases, and tumor immune responses, such interactions may have important biological significance and therapeutic potential.

### Exploration of cGAS–STING axis as a potential therapeutic target for neuroinflammation

Inter-individual differences and precision medicine: Given the accumulation of information relating to individual genomes, future research may focus on cGAS–STING axis differences between individuals. Understanding how these differences affect disease progression and therapeutic outcomes will be key to enabling personalized medicine.

Development of new therapeutic agents: Research will drive the development of new therapeutic agents that more precisely modulate cGAS–STING axis, whether by inhibiting it to treat inflammatory diseases, or by activating it to enhance antiviral and antitumor immune responses. The translation of fundamental laboratory discoveries into clinical applications necessitates further preclinical and clinical investigations to evaluate the feasibility of targeting cGAS–STING axis in patient populations.

Application of smart technologies: Future research endeavors could leverage artificial intelligence and machine learning techniques to analyze extensive datasets and predict the activation patterns of cGAS–STING axis, along with elucidating its specific roles in various diseases.

As a therapeutic target for neuroinflammation, cGAS–STING axis holds significant promise for application and is expected to be optimistic. Future studies may identify new therapeutic approaches that utilize this axis to treat neurodegenerative, autoimmune, and neuroinflammatory diseases. As our understanding of this axis increases, new drugs and treatments will emerge, bringing new hope to patients with neurological disorders.

## Limitations

Within the realm of neuroinflammation, the activation of the cGAS–STING axis not only induces the production of interferons and other inflammatory mediators but also has the potential to inflict damage on neural cells. This phenomenon demonstrates considerable spatiotemporal specificity across various neurological disorders. For instance, in cases of acute nerve injury, activation of the cGAS–STING axis may facilitate the initial immune response, thereby aiding in tissue repair. Conversely, in scenarios of chronic neuroinflammation, sustained activation can result in neuronal apoptosis and dysfunction. Consequently, the challenge of balancing the beneficial and detrimental effects of this pathway has emerged as a critical focus of contemporary research.

## Conclusions

The cGAS–STING axis is integral to the regulation of neuroinflammation, with its dysregulation being significantly implicated in the pathogenesis of various neurodegenerative diseases and neuroinflammatory conditions, including herpes simplex encephalitis, AD, MS, PD, AT, and HD. The pathway’s spatiotemporal dependence is evident as its mechanisms of action differ across patients’ disease states and stages of disease progression. This intricate regulatory feature not only enhances our understanding of neuroinflammation but also offers novel insights for clinical interventions. Recent research has demonstrated that the cGAS–STING axis exerts a dual role in modulating immune responses and providing neuroprotection, thereby establishing a foundation for the development of therapeutic strategies targeting neuroinflammation.

Future advancements in spatiotemporal-specific regulatory tools are anticipated to be crucial in addressing this issue. By precisely modulating the timing and intensity of activation within the cGAS–STING axis, it may become possible to more effectively manage the progression of the condition during the initial phases of the inflammatory response. This approach aims to promote neuroprotection while simultaneously mitigating hyperactivity in chronic conditions. Such a strategy not only enhances the therapeutic efficacy in managing neuroinflammation but also has the potential to minimize adverse effects, thereby offering patients a safer and more effective treatment option. Simultaneously, investigations into the cGAS–STING axis offer profound insights into the integration of diverse perspectives and findings when addressing complex disease mechanisms. The use of varied experimental models and research methodologies can yield differing interpretations of the same pathway, underscoring the necessity for an open-minded approach during analysis and the importance of fostering interdisciplinary collaboration and dialogue. By synthesizing fundamental research with clinical applications, we can achieve a more comprehensive understanding of the nature of neuroinflammation and advance innovation in related therapeutic strategies.

Future research should not only emphasize the development of spatiotemporal specificity regulatory tools but also concentrate on elucidating the interactions between the cGAS–STING axis and other signaling pathways. Such a multidimensional approach will enhance our understanding of the complexity of neuroinflammation and identify more effective targets for clinical intervention. This research trajectory will establish a foundation for a more profound comprehension of the mechanisms underlying neuroinflammation and the advancement of novel therapeutic strategies. Given the significance of individual variability in the onset and progression of neurological disorders, devising personalized treatment strategies will also constitute a crucial avenue for future investigation.

In conclusion, research on the cGAS–STING axis in neuroinflammation not only introduces novel concepts and strategies but also fosters the interdisciplinary integration of neuroscience and clinical medicine. A comprehensive understanding of its spatiotemporal dynamics, coupled with the development of precise therapeutic interventions, holds the potential to significantly advance the treatment of neuroinflammation. Continued investigation in this domain is anticipated to yield critical scientific insights and practical guidance, facilitating the attainment of equilibrium between neuroprotection and immune homeostasis.

## Data Availability

*Not applicable*.
